# Current evidence and future perspectives in the exploration of sleep-related eating disorder–a systematic literature review

**DOI:** 10.3389/fpsyt.2024.1393337

**Published:** 2024-05-30

**Authors:** Octavian Vasiliu

**Affiliations:** Department of Psychiatry, Dr. Carol Davila University Emergency Central Military Hospital, Bucharest, Romania

**Keywords:** sleep-related eating disorder, night eating syndrome, parasomnia, disorders of arousal, topiramate, clonazepam, pramipexole, zolpidem

## Abstract

Sleep-related eating disorder (SRED) is a non-REM parasomnia with potentially significant negative effects on general health (dangerous activities during night eating episodes, obesity, or metabolic syndrome, for example). Although the history of SRED encompasses more than six decades, public awareness and even the awareness of the mental health specialists of this disorder is very limited, a phenomenon that hinders the development of research in this field. Therefore, a systematic review based on PRISMA 2020 guidelines explored the available evidence for SRED found in four electronic databases (PubMed, Cochrane Collaboration, Google Scholar, and Clarivate/Web of Science). A number of 94 primary and secondary reports were retrieved, investigating aspects regarding the risk factors, epidemiology, clinical data and differential diagnosis, epidemiology, structured evaluation, and treatment of SRED. Based on the results of these reports, Z-drugs, but also certain benzodiazepines, antidepressants, antipsychotics, and psychostimulants may trigger the onset of SRED. Psychiatric and neurologic disorders have also been associated with SRED, either as risk factors or comorbid conditions. Cerebral glucose metabolism dysfunctions, neurotransmitter dysfunctions, and genetic factors have been invoked as pathogenetic contributors. Structured assessment of SRED is possible, but there is a dearth of instruments dedicated to this purpose. Data on the prevalence and treatment of SRED exist, but good-quality epidemiological studies and clinical trials are still missing. In conclusion, future research is expected to address the shortcomings of SRED exploration by creating the conditions for better quality and larger group clinical research. The need for such investigation is granted by the importance of this pathology and its negative functional consequences.

## 1 Introduction

A dysfunction of eating behavior stands as the core criterion for many clinical entities, such as anorexia nervosa, bulimia nervosa, food addiction, binge eating disorder (BED), pregorexia nervosa, anorexia athletica, bigorexia, etc. (1–3). Also, eating behavior dysfunctions may be reported in patients diagnosed with disorders from different nosological categories, such as depressive disorders, schizophrenia spectrum disorders, neurocognitive disorders, or anxiety disorders (1–4). Each eating disorder, either well-supported by evidence and included in one or more of the international classifications or still undergoing investigation to find evidence to confirm its existence, has a specific profile of eating behavior, clinical consequences, impact on self-image, or presumed pathophysiology. Also, the therapeutic approach to these eating disorders is quite different, based on specific clinical manifestations, high rate of comorbid conditions, and vulnerability, as well as on various precipitating factors (4–7). It is estimated that more than 3.3 million healthy life years worldwide are lost each year due to eating disorders, and the number of years lived with disability (YLDs) in patients with anorexia nervosa and bulimia nervosa is increasing (8). Also, patients who were hospitalized for anorexia nervosa have a more than five times increased mortality risk, according to the same source. To further complicate the overall picture of eating disorders’ consequences, the quality of life is decreased in these patients, yearly healthcare costs are significantly higher than in the general population, the number of offspring is reduced, and risks for pregnancy complications are increased (8). Patients with anorexia nervosa often experience social maladjustment and physical problems, especially if the duration of the disorder is prolonged (9). Not only do patients with eating disorders suffer important consequences, but also their caregivers reported reduced income and productivity, higher daily costs, and significant psychosocial impacts on family life, interpersonal relationships, and their own personal well-being (10).

When referring to parasomnias, the discussion about clinical criteria, pathophysiology, and treatment is similar, because this is an extremely heterogeneous category that includes disorders associated with REM sleep, non-REM sleep, and other types, as well as isolated symptoms or normal variants, all requiring tailored diagnostic and therapeutic approaches (11).

According to the 5th edition (revised) of the Diagnostic and Statistical Manual of Mental Disorder (DSM-5TR), non-REM sleep arousal disorders (NREMSADs) have as potential specifiers (1) „sleepwalking type”, with subsequent specifiers „sleep-related eating” and „sleep-related sexual behavior (sexsomnia)”, or (2) „sleep terror type” (12). Sleepwalking episodes are observed most commonly during slow-wave sleep (SWS), usually occurring in the first third of the night (12). Sleep-related eating disorder (SRED) is defined by the onset of recurrent episodes of abnormal eating behaviors during an incomplete awakening and it may involve simple or complex motor behaviors, starting from grabbing some snacks or inedible items placed near the bed, to cooking meals, or driving to the shop for purchasing food (12, 13). All eating behaviors reported during sleepwalking episodes are associated with amnesia (either partial or complete) and these individuals may present themselves with weight gain due to food ingestion during the night and, also, they may have injuries secondary to the involuntary consumption of inedible items (13). Also, clinically significant distress or dysfunctions in social, occupational, and other areas of daily functioning are associated with all NREMSADs, according to DSM-5TR (12).

NREMSADs, SRED included, are considered parasomnias by the latest DSM version, and therefore clustered in the same large nosological category as “nightmare disorder” and “REM-sleep behavior disorder” (RBD), based on similar clinical features, such as „abnormal behavioral, experiential, or physiological events” that occur during sleep or sleep-wake transitions (12). However, other authors consider that SRED and related disorders (i.e., sleepwalking, sexsomnia, sleep terrors, confusional arousals, and sleep-related choking syndrome) are part of the nosological category named „disorders of arousal” (DOAs), based on epidemiological (e.g., a similar prevalence in children and adults, and possible familial aggregation) and clinical (e.g., risk of self-injury, automatic behaviors, partial/complete amnesia, excessive daytime sleepiness, pain, and altered quality of life) data (14).

Also, the detection of a local arousal pattern in motor and limbic regions, coupled with preserved or even increased intensity of sleep wave patterns in the frontoparietal region, suggested a dissociated model of brain activity, according to the electroencephalography (EEG) and functional brain imaging explorations (14–18). Several parasomnias, such as RBD, have been related to the possibility of predicting neurological disorders, such as Parkinson’s disease (17, 19). The quality of sleep may be severely impacted by a broad spectrum of disorders, both organic and psychiatric, and exploring these potential causes, when the patients accuse insomnia, daytime somnolence, fatigability, asthenia, or difficulties in concentration, is necessary to construct adequate case management for these patients. Between these disorders, bruxism, temporomandibular disorders, and pathologies associated with chronic pain are worthy of special exploration due to their severe impact not only on sleep quality but also on overall functionality, quality of life, and general well-being (20–25). The second edition of the International Classification of Sleep Disorders (ICSD-2) defined SRED as „recurrent episodes of involuntary eating and drinking” with onset during the main sleep period, presenting one or more criteria within the following list: strange or unusual foods, even inedible or toxic compounds; insomnia, nonrestorative sleep, daytime fatigue, somnolence; sleep-related injuries; dangerous behaviors during the episodes of eating; morning anorexia; negative impact of these episodes on personal health. The exclusionary criterion implies the differentiation of SRED from other sleep disorders, medical or neurological diseases, drug-related disorders, or eating disorders (26). According to ICSD-2, SRED is included in the category of „other parasomnias”, together with sleep-related dissociative disorders, sleep enuresis, sleep-related groaning (or catathrenia), exploding head syndrome, sleep-related hallucinations, unspecified parasomnia, and parasomnias due to drugs or medical conditions (26, 27).

In the third edition of ICSD, the core criterion was rephrased as „repeated non-adoptive food intake after waking up during the main sleep period”, and the list of associated symptoms was shortened to three criteria: uneatable food or poisonous substances consumed during these episodes; damage or possible damage during the cooking processes or searching-for-food behaviors; negative health consequences (11). Additionally, there is a criterion referring to a complete or partial loss of consciousness during the episodes, followed by impaired memory. The exclusionary criterion was preserved in the third edition of ICSD (11). Unlike the previous edition, in ICSD-3, SRED is included in the section of „non-REM-related parasomnias”, which may signal not only the need to increase the compatibility of this classification with the DSM system but also a tendency to integrate more accurately SRED in the configuration of parasomnias.

The latest edition of the International Classification of Diseases (ICD-11) does acknowledge the existence of SRED, as part of the „Disorders of arousal from non-REM sleep”, and defines this entity by the occurrence of „recurrent episodes of involuntary excessive or dangerous eating or drinking (…) during the main sleep period” (28). ICD-11 also mentions the consumption of inedible, toxic, or peculiar foods/substances/combinations of foods, as well as the possibility of injurious behaviors while searching for food or cooking. The negative impact of recurrent nocturnal episodes consisting of consuming high-calorie foods on individual health is another diagnosis criterion, as is the partial/complete amnesia for these events (28). This approach to SRED is a significant change compared to the previous edition, ICD-10, which did not define this clinical entity but included it in the residual category of „other sleep-related movement disorders” (29).

From a historical perspective, the first time abnormal eating behaviors during the nighttime were described in the literature in a systematic manner was in 1955, when these behaviors were labeled „night-eating syndrome” (NES), after their detection in obese patients (30). However, this concept refers only to conscious, dysfunctional eating behaviors during the night, thus contradicting the criteria of SRED, which may be considered as diagnosis only when such behaviors occur during sleep or sleep-wake transitions, as mentioned before. Therefore, the first communication about nocturnal eating behaviors reported in sleeping individuals can be considered the seminal work of Ekbom, who published in 1960 an article on restless legs syndrome (RLS) (31, 32). Ekbom reported that nocturnal eating behaviors were observed in patients with RLS and associated with impaired sleep (31). Further research on this topic showed that „involuntary, nocturnal, sleep-related eating” is often accompanied by other dysfunctional behaviors during the night, and in a 5-year study (N=19 participants, 14 female and 5 male) this condition was described as having a chronic evolution, high frequency of symptoms (nightly in 58% of the patients) and severe negative consequences (33). Excessive weight gain, worries about choking during meals or about starting fires from cooking, and poor sleep quality were described in these cases (33). In this latest study, which may be considered the first research strictly targeting SRED, only two patients presented a daytime eating disorder (anorexia nervosa), but more than 47% of the participants had an Axis I psychiatric diagnosis (mostly mood and anxiety disorders) (33). Previously, in 1990, in a case series (N=3 patients), Whyte & Kavey reported the existence of “somnambulistic eating”, documenting on polysomnography the occurrence of episodic eating behaviors in non-REM sleep, but these patients also complained of conscious nocturnal eating episodes (34).

Based on these preliminary, historical data, SRED can be considered a specific pathology, and its existence is acknowledged by all the current major nosological classifications (11, 12, 26, 28). However, several aspects still require detailed analysis because (a) the awareness of this disorder in the general population, but also its recognition by general practitioners and even mental health specialists, is reduced, and this phenomenon can negatively impact the process of diagnosing such pathology, finding its real incidence and prevalence, initiating dedicated clinical trials, etc.; (b) although this disorder has a quite long history (more than six decades, if we consider the previously mentioned article of Ekbom as the starting point, or more than 30 years if we choose the work of Schenk et al. (1991) as the main reference), its pathophysiology is poorly understood, and the dual nature of this disorder (eating disorder and parasomnia) raises interesting questions about the mechanisms of its onset and development; (d) due to the severe functional consequences of SRED, evidence-based recommendations for early detection and treatment are needed.

This systematic review, based on PRISMA 2020 guidelines (**Supplementary Table S1**) (35), has as its objective the identification of relevant data regarding the risk factors, differential diagnosis, epidemiology, pathophysiology, structured clinical evaluation, and treatment of SRED.

## 2 Methodology

Four major electronic databases (PubMed, Cochrane-, Clarivate/Web of Science, and Google Scholar) were searched using the paradigm “sleep-related eating disorder” AND “epidemiology” OR “diagnosis” OR “pathophysiology” OR “risk factors” OR “questionnaires” OR “inventories” OR “test” OR “treatment”. Also, the lists of references for each retrieved article that reached the second phase of the review were manually searched for possibly relevant reports. No language restrictions were implemented, and all types of available sources were reviewed, i.e., both primary and secondary reports. The search interval was between the inception of each electronic database up to August 2023.

The main inclusion criteria were (1) the presence of SRED as the main diagnosis; (2) all types of research, from case reports and case series to clinical trials and meta-analyses; (3) inpatient and outpatient studies were allowed; (4) the outcomes referred to epidemiological, pathophysiological, clinical, and therapeutic variables; (5) any type of intervention was permitted; (6) no restriction regarding the language of publication was applied. The core exclusion criteria were (1) unspecified criteria for SRED diagnosis; (2) unclear methodology or environment of the research; (3) other outcomes than those already specified; (4) insufficiently defined design of the research. The complete list of inclusion and exclusion criteria is presented in **Table 1**.

**Table 1:** Inclusion and exclusion criteria.

Operational criteria	Inclusion criteria	Exclusion criteria
**Population**	All age groups were allowed. The main diagnosis explored was “sleep-related eating disorder”. Diagnoses made according to the DSM, ICD, or ICSD nosographic systems (no limitations regarding the edition) were permitted, but also original criteria constructed by authors of the respective reports.	Unspecified diagnoses or reports that included various EDs or sleep disorders without clarifying what criteria were used during the research.
**Intervention**	Any type of study, such as clinical or preclinical research, epidemiological or clinical, prospective or retrospective, etc. Any type of review, such as systematic, narrative, scoping, meta-analysis, umbrella review, etc.	Studies with undetermined methodology and reviews with unspecified design.
**Environment**	Inpatient, outpatient, daycare, and general population.	Unspecified environment.
**Primary and secondary variables**	Prevalence, incidence, risk factors, clinical diagnosis, pathophysiological data, psychological evaluation, and treatment	Imprecisely defined or poorly characterized variables, and reports without pre-defined outcomes.
**Study design**	Primary and secondary reports, clinical and preclinical research.	Unspecified or insufficiently defined designs.
**Language**	Any language of publication was admitted.	

ED, eating disorder; DSM, Diagnostic and Statistical Manual of Mental Disorders; ICD, International Classification of Diseases; ICSD, International Classification of Sleep Disorders.

The overall quality of data (OQD) assessment was based on Joanna Briggs Institute’s (JBI) critical appraisal checklists for quantitative and qualitative research (36–41). These tools were chosen because it was expected, due to the objective and inclusion/exclusion criteria of this review, that sources would be heterogeneous, varying from case reports and case series to clinical trials, and from epidemiological studies to literature reviews and meta-analyses. Scores for OQD were given based on the criteria met by each source, then transformed into one of the following four categories: “very low” (less than 25% of the criteria met), “low” (26–50%), “moderate” (51–75%), and “high” (76–100%).

## 3 Results

**Figure 1:**
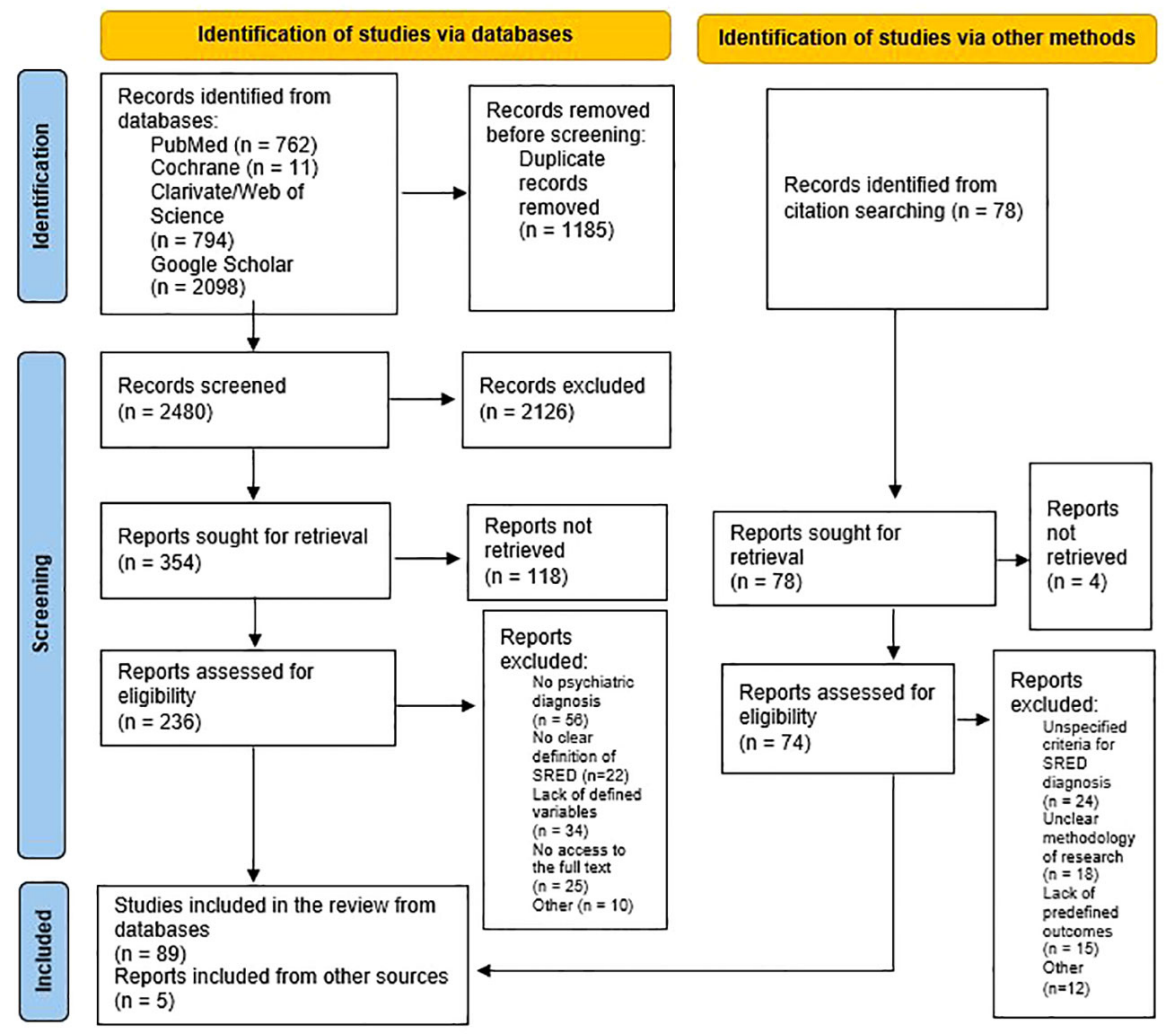
PRISMA flow diagram for searching of databases and other sources (35).

After applying the search paradigm, a total number of 3665 papers were found, but after deduplication, only 2480 remained (**Figure 1**). The application of the inclusion/exclusion criteria led to the preservation of 89 out of the screened papers, which entered the final phase of the review. Another 78 references were explored after the lists of references were consulted, but only 5 were considered for review after pre-defined selection criteria were applied (**Figure 1**).

Therefore, a total number of 94 *in-extenso* papers were reviewed in detail, representing 50 reports on risk factors, 13 on comorbidities, 18 on pathogenesis, 14 on differential diagnosis, 10 on epidemiological data, 5 on structured evaluation, and 30 on treatment (with a degree of overlap between sources depending on the pre-determined outcomes) (**Tables 2**–**4**). The OQD for each research is also presented in the corresponding tables, and most reviewed sources were evaluated as presenting low and very low quality (**Supplementary Table S2**).

**Table 2:** Reports included in the review referring to the drug-associated SRED behaviors.

Reference	Type of paper	Main outcomes	Results and observations	OQD
(42)	Review (N=148 patients)	Incidence of drug-induced SRED	Zolpidem-induced complex sleep behaviors (N=79 patients from case reports and case series, N=69 patients from 1454 patients treated with zolpidem in three observational clinical studies); 88% of cases were found to be probably associated with zolpidem	Low
(43)	Case series (N=2 Malay women)	Evolution of drug-induced SRED	Quetiapine may induce SRED at various doses, ranging from 50 to 200 mg/day	Low
(44)	Review	Evolution of drug-induced SRED	Triazolam, lithium, olanzapine, risperidone, zopiclone, zaleplon, and zolpidem ER may be associated with new-onset SRED cases	Low
(45)	Review (n=10 reports, N=17 patients)	Onset of SRED and other sleep-related behaviors	Zolpidem>zopiclone, zalepon	Very low
(46)	Retrospective study (N=676 AE reports)	Drug-associated SRED cases	Zolpidem (36%)>sodium oxybate (27%)>quetiapine (14%); aripiprazole may be associated with SRED episodes (3.6%); SNRIs antidepressants also determined SRED episodes (2.7% for duloxetine, 2.1% for venlafaxine); psychostimulants (0.4-1.5%) may associate new onset SRED cases	Moderate
(47)	Retrospective study (N=5784 AE reports)	Drug-associated SRED and somnambulism	508 SRED cases out of 5784 reports of SRED and somnambulism; quetiapine also was associated with SRED in >53% of these reports	Moderate
(48)	Cross-sectional study (N=1318 patients taking hypnotics)	Drug-associated SRED	8.4% presented new-onset SRED, especially young subjects, ↑doses of DZP-equivalent doses, ↑PSQI scores	Moderate
(49)	Retrospective study (n=125 patients)	Sleep-related behaviors in patients with MDD, anxiety disorders, adjustment disorders, somatoform disorders, or sleep disorders treated with hypnosedatives	~15% presented complex sleep-related behaviors, all were treated with zolpidem (over 10 mg/day)	Moderate
(50)	Review (n=40 case reports)	SRED onset	Zolpidem (≥10 mg/day) use was associated with SRED	Moderate
(51)	Case series (N=8 patients)	SRED onset in patients treated with zolpidem	Zolpidem triggered SRED behaviors (1-8 episodes/night)	Low
(52)	Case report (Caucasian woman, 53-year-old)	SRED onset and evolution	Zolpidem-induced SRED episodes (2-3 episodes/week) and weight increase (6 kg after 12 months)	Moderate
(53)	Case report (African-American woman, 51-year-old)	SRED onset and evolution	Zolpidem IR triggered the onset of sleepwalking, SRED, and sleep-driving	Low
(54)	Case series (N=5 patients)	Nocturnal eating behaviors	Zolpidem determined SRED and these behaviors disappeared after the drug’s discontinuation	Low
(55)	Case report (a 45-year-old man)	SRED behaviors	Zolpidem determined night eating and cooking activities	Low
(56)	Case report (a 46-year-old woman)	SRED behaviors	Zolpidem was administered for insomnia and induced amnestic nocturnal eating behaviors. Switching to eszopiclone led to the complete remission of SRED.	Moderate
(57)	Case report (a 45-year-old man)	SRED onset and evolution	Sleepwalking and nocturnal eating behaviors followed by complete amnesia appeared after zolpidem CR was administered; symptoms disappeared after stopping zolpidem use	Low
(58)	Case report (a 71-year-old Korean man)	SRED onset and evolution	Zolpidem CR triggered SRED and other sleep-related complex behaviors; these symptoms disappeared once zolpidem was stopped	Moderate
(59)	Case report (a 49-year-old man)	SRED onset and evolution	Lamotrigine + clonazepam + zolpidem was the combination used to treat this patient with BD; SRED behaviors appeared after the initiation of zolpidem and disappeared when this drug was discontinued	Low
(60)	Case report (a 21-year-old woman)	SRED onset and evolution	This patient was diagnosed with ADHD and zolpidem was associated with SRED behaviors, which disappeared after this drug’s discontinuation and replacement by clonazepam	Very low
(61)	Case series (N=2 patients)	SRED onset and evolution	Zolpidem ER 12.5 mg/day led to amnestic night-eating behaviors; switching on zolpidem IR led to the remission of these behaviors	Low
(62)	Case report (a 49-year-old woman)	SRED onset and evolution	This patient was diagnosed with MDD and received treatment with duloxetine and zolpidem up to 15 mg/day; SRED appeared and a switch on zaleplon 10 mg/day was initiated, but SRED and NES episodes persisted; zaleplon discontinuation led to the remission of night eating behaviors	Moderate
(63)	Case report (a 48-year-old Japanese woman)	SRED onset and evolution	Triazolam administration led to SRED behaviors; a dose decrease was followed by a reduced frequency of SRED episodes	Moderate
(33)	Case series (N=19 patients)	SRED onset and evolution	Nocturnal eating appeared immediately after triazolam abuse, and its discontinuation led to symptoms’ remission; amitriptyline (200 mg/day) caused might-eating behaviors that disappeared after the drug’s discontinuation	Moderate
(64)				Moderate
(65)	Case report (a 9-year-old boy)	SRED onset and evolution	This patient was diagnosed with severe ADHD, clonus dystonia, and insomnia, and clonazepam (0.5 mg/day) was initiated; SRED appeared rapidly after clonazepam administration, and the discontinuation of this drug led to complete SRED remission	Low
(66)	Case report (a 42-year-old man)	SRED onset and evolution	Sodium oxybate (4.5-8 g/night) initiated for narcolepsy-cataplexy led to the onset of complex activities during sleep, SRED included; these symptoms disappeared after the dose was reduced to 7 g/night	Low
(67)	Case report (a 51-year-old woman)	SRED behaviors in a patient with schizophrenia	Haloperidol determined RLS, SRED and NES	Low
(68)	Case report (a 52-year-old man)	SRED in a patient with type I BD	Olanzapine (10 mg/day) added to lithium was responsible for sleepwalking and nocturnal eating episodes with complete amnesia; olanzapine’s discontinuation reversed these episodes	Low
(69)	Case report (a 41-year-old Japanese man)	SRED onset in a patient with MDD	Aripiprazole (10 mg/day) added to sertraline led to the onset of SRED episodes; reducing the dose to 1.5 mg/day led to the rapid and complete remission of night eating behaviors	Low
(70)	Case report (a 48-year-old woman)	SRED in a patient with rapid-cycling BD	Quetiapine at bedtime (100 mg) led to the onset of somnambulism and nocturnal eating followed by amnesia	Low
(71)	Case series (N=2 patients)	SRED onset in patients with OSA	Quetiapine-induced sleepwalking and SRED-like behaviors; quetiapine discontinuation + CPAP therapy led to these symptoms remission	Low
(72)	Case report (a 68-year-old man)	SRED onset in a patient with vascular dementia+ psychotic symptoms	Risperidone (2 mg/day) determined the onset of nocturnal eating behaviors + complete amnesia; these symptoms disappeared when the dose decreased to 1 mg/day	Low
(73)	Case report (a 16-year-old girl)	SRED onset and evolution	Risperidone (1 mg/day) led to the onset of SRED behaviors, including dangerous cooking activities; after risperidone was stopped, these eating behaviors disappeared	Low
(74)	Case report (a 28-year-old white male)	SRED onset in a patient with schizoaffective disorder	Ziprasidone (120 mg/day) induced sleepwalking and SRED; decreasing the dose to 40 mg/day led to the disappearance of SRED; re-challenging with 120 mg/day led to the re-appearance of SRED.	Low
(75)	Case report (a 24-year-old woman)	SRED onset in a patient with MDD	Fluoxetine (40 mg/day) + trazodone (75 mg/day) + zolpidem (10 mg/day) triggered episodes of nocturnal binge eating with amnesia; switching to mirtazapine (30 mg/day) and clonazepam (0.25 mg/day) led to the transient remission of SRED, but only the complete discontinuation of this antidepressant allowed for the disappearance of SRED	Low
(76)	Case report (a 19-year-old woman)	SRED onset in a patient with anxiety, depressed mood, and suicidal ideation	Mirtazapine (30 mg/day) led to the development of SRED episodes; these manifestations remitted when the dose was decreased to 15 mg	Low
(77)	Case report (a 33-year-old white man)	SRED in a patient with nicotine use disorder	Bupropion SR (300 mg/day) induced nocturnal eating episodes, sleepwalking, and telephone use with partial/complete amnesia; these episodes disappeared after the antidepressant’s discontinuation	Low
(78)	A case-control study (N=100 patients with RLS and 100 matched controls)	SRED onset in patients with RLS	A trend toward the association of dopaminergic agents or hypnotic drugs with SRED in this population was reported (p=0.20)	Moderate
(79)	Expert opinion	SRED in patients with RLS or PD	L-dopa/carbidopa and bromocriptine may be associated with new-onset SRED cases	Low

AE, adverse event; BD, bipolar disorder; CPAP, continuous positive airway pressure; CR, controlled release; DZP, diazepam; ER, extended release; IR, immediate release; MDD, major depressive disorder; NES, night eating syndrome; OQD, overall quality of data; OSA, obstructive sleep apnea; PD, Parkinson’s disease; PSQI, Pittsburg Sleep Quality Index; RLS, restless legs syndrome; SR, sustained release; SRED, sleep-related eating disorder.

**Table 3:** Reports included in the review referring to non-pharmacological triggers of SRED behaviors.

Reference	Type of paper	Main outcomes	Results and observations	OQD
(33)	Prevalence studies (N=19 and 700 participants, respectively)	Risk factors for SRED	Depression severity, dissociative symptoms, SUDs; daytime eating disorder, NES, other sleep disorders	Moderate
(80)				Moderate
(64)	Case series (N=19 patients)	Risk factors for SRED (pharmacological and non-pharmacological)	OSA, PLM, familial sleepwalking, and irregular sleep/wake pattern disorder, familial RLS, anorexia nervosa with nocturnal bulimia, and migraines treated with amitriptyline were associated with SRED and NES behaviors; acute stress derived from worries about the safety of family members or relationships problems may trigger SRED	Moderate
(44)	Narrative review	Risk factors for SRED	Other sleep disorders can be considered a risk factor for SRED	Low
(32)	Survey-based study (N=130 patients)	Risk factors for NES and SRED	RLS is frequently related to SRED, possibly mediated by mistreatment with sedative agents	Low
(50)	Review (n=40 case reports)	Risk factors for SRED	OSA, MDD, and RLS were the most frequent disorders reported in patients with SRED	Moderate
(81)	Survey-based study (N=53 patients)	Risk factors for SRED in a group of patients with sleep disorders	66% of the responders had frequent night-eating behaviors, 45% had SRED	Very low
(78)	A case-control study (N=100 patients)	Risk factors for SRED in patients with RLS	RLS was associated more frequently with SRED than the control group; ↑MOCI scores in patients with both RLS and SRED	Moderate
(82)	Cross-sectional study (N=120 patients)	SRED and NES in patients with RLS	SRED or NES were detected more frequently in patients with RLS than in the general population	High
(83)	Case report (a 34-year-old white man)	SRED onset and evolution	OSA and SRED can be frequently detected together; OSA may precipitate the onset of SRED	Low
(84)	Case series (N=2 patients)	SRED onset and evolution	Narcolepsy and OSA may be predisposing factors for SRED; work-related stress, disturbance of the circadian rhythm due to professional tasks, and insufficient time allocated to sleep were reported as risk factors for SRED	Low
(85)	Cross-sectional study (N=65 patients)	SRED in patients with narcolepsy vs. healthy controls	Narcolepsy and cataplexy were more frequently associated with SRED; ↑severity of depression, in females, and higher scores of bulimia and social insecurity on EDI-2, ↑MOCI scores	Moderate
(86)	Controlled study (N=36 patients)	SRED vs. sleepwalking profiles	A personal history of eating problems in childhood and ↑current anorexia scores were reported in patients with SRED/sleepwalking vs. healthy controls	Moderate
(87)	A case series (N=2 patients)	SRED in patients with PD	OSA, NES, and REM sleep disorders were present as comorbidities;	Low
(88)	Case report (a 56-year-old woman)	SRED in a patient with PD	SRED was detected together with PD, OSA, sleepwalking, depression, and REM sleep parasomnia	Low

EDI-2, Eating Disorder Inventory-2; SUD, substance use disorders; MDD, major depressive disorder; MOCI, Maudsley Obsessive-Compulsive Inventory; NES, night eating syndrome; OQD, overall quality of data; OSA, obstructive sleep apnea; PD, Parkinson’s disease; PLM, periodic limb movements; RLS, restless legs syndrome; SRED, sleep-related eating disorder.

**Table 4:** Reports included in the review referring to the treatment of SRED behaviors.

Reference	Type of paper	Main outcomes	Results and observations	OQD
(13)	Expert opinion	The efficacy of pharmacological interventions in patients with SRED	Pramipexole was efficient in SRED + RLS cases; sleepwalking + SRED may benefit from low doses of clonazepam; regular follow-up is recommended for all patients with SRED at least 2-3 times/year; first-line treatment for SRED includes SSRIs, with topiramate and clonazepam as alternative	Low
(14)	Expert opinion	The efficacy of treatments for DOAs	Removal of precipitating factors and prevention	Low
(33)	Case series (N=19 patients)	Clinical evolution and polysomnographic data in SRED patients undergoing various therapeutic approaches	Adequate treatment of comorbid disorders and vulnerabilities	Moderate
(63)	Case report (a 48-year-old Japanese woman	Evolution of SRED symptoms during treatment	Pramipexole 0.125 mg + clonazepam improved SRED, RLS, and sleepwalking	Moderate
(67)	Case report (a 51-year-old woman)	Evolution of SRED symptoms during treatment	Clonazepam completely eliminated the RLS episodes and nocturnal eating	Low
(64)	Case series (N=19 patients) + two reviews	Evolution of SRED symptoms during various therapeutic interventions	CPAP for SRED + OSA, evidence is sparse; fluoxetine was efficient; targeting the primary sleep disorder is essential; carbidopa/l-dopa, bromocriptine +/- codeine in SRED + sleepwalking or PLM	Moderate
(89)				Low
(90)				Low
(83)	Case report (a 34-year-old white man)	Effect of limited offering of food during nighttime	The effect of this intervention was favorable	Low
(84)	Case series (N=2 patients)	SRED evolution during pharmacological treatment	Sertraline (25 mg/day) induced SRED symptoms’ remission	Low
(91)	Controlled trial (N=11 patients)	The efficacy of pramipexole on clinical and actigraphic parameters in patients with SRED	Pramipexol (0.18-0.36 mg/day) was efficient in decreasing the median night duration of SRED; the tolerability was good	Moderate
(92)	Case report (a 29-year-old man)	Effect of limited offering of food before going to bed	The effect of this intervention was favorable	Low
(93)	Review + expert opinion	Considerations on the treatment of NES and SRED	None of the explored treatment options for NES/SRED had long-term efficacy in good-quality trials	Low
(94)	Case series (N=7 cases)	Pharmacological treatment of SRED	Dopaminergic + opioid agents +/- sedative agents prn were efficient	Low
(95)	Case report (a 35-year-old Caucasian man)	The efficacy of pharmacological treatment in a patient with obesity and SRED	Phentermine + topiramate ER was well tolerated and efficient	Low
(96)	RCT (N=34 patients)	SRED evolution during treatment with topiramate vs. placebo	Topiramate (up to 300 mg/day) decreased the episodes of SRED	Very high
(97)	Clinical study (N=17 patients)	The efficacy and tolerability of topiramate in the treatment of SRED	Topiramate was efficient and well-tolerated	Low
(98)	Case series (N=4 patients)	SRED and NES evolution during topiramate treatment	Topiramate was efficient at doses of 100 mg/day	Low
(99)	Case report (a 45-year-old woman)	Efficacy of topiramate in a patient with sleepwalking, SRED, sleep-related smoking, and mild OSA	Topiramate (100 mg/day) led to the complete resolution of dysfunctional nocturnal behaviors	Low
(100)	Retrospective chart review (N=30 patients)	SRED evolution determined by CGI-I scores during topiramate treatment	68% were responders after 11.6 months of treatment; AEs were reported by 84% of the participants, and 40% discontinued the treatment	Moderate
(101)	Case report (a 28-year-old man)	SRED and sleepwalking symptoms evolution during pharmacological treatment	Clonazepam (2 mg/day) + fluoxetine (20 mg/day) failed to control sleep-related behaviors, but topiramate (50 mg/day) was successful; the tolerability was good	Low
(102)	Case series (N=4 patients)	The effects of SSRIs on the nocturnal eating/drinking disorder	Fluvoxamine and paroxetine were efficient for SRED symptoms	Low
(103)	Case report (a 54-year-old white woman)	The effects of antidepressants on SRED	Agomelatine controlled sleep-related eating symptoms, but when the drug was discontinued, SRED symptoms re-appeared	Moderate
(104)	Case series (N=2 patients)	Efficacy of treatment in patients with SRED, monitored with polysomnography	The combination of bupropion + l-dopa + trazodone led to good results in patients with SRED	Low
(105)	Case report (a 25-year-old woman)	Efficacy of treatment orexin antagonists in SRED	Suvorexant was efficient in a patient diagnosed with depression and SRED	Moderate
(106)	Retrospective study (N=49 patients)	Efficacy of pharmacological treatment in patients with SRED	Ramelteon (4-8 mg/day) as an add-on to the ongoing benzodiazepine treatment was followed by a dose reduction of benzodiazepine and this was an efficient strategy	Very high
(107)	A 5-year follow-up study (N=36 patients, adults and children)	Efficacy of hypnotherapy (two sessions) in patients with parasomnias	Only two patients presented SRED, but the overall rate of response was good	Low
(108)	Case report (a 38-year old woman)	Efficacy of hypnotherapy in a patient with SRED and sleepwalking	The episodes of SRED/sleepwalking decreased significantly	Low
(109)	Retrospective study (N=46 patients) + a literature review of nonpharmacological treatments for parasomnias	The efficacy of five outpatient CBT-NREMP sessions	CBT-NREMP was efficient in decreasing the severity of NREM parasomnia, insomnia, and anxiety and depression severity; the significance for SRED is uncertain due to the low representation of this pathology in the study sample (1.6%)	Moderate
(110)				Moderate

AE, adverse events; CBT, cognitive-behavioral therapy; DOAs, disorders of arousal; NES, night eating syndrome; NREMP, NREM parasomnias; CGI-I, Clinical Global Impressions of Improvement; OQD, overall quality of data; OSA, obstructive sleep apnea; PLM, periodic limb movements; prn, pro re nata (as needed); RCT, randomized controlled trial; RLS, restless legs syndrome; SRED, sleep-related eating disorder; SSRIs, selective serotonin reuptake inhibitors.

### 3.1 Risk factors for SRED onset

These risk factors have been distributed in three distinct categories, i.e., pharmacological triggers, psychiatric and organic predisposing or precipitating conditions, and other factors. Although the causal relationship between these factors and SRED is not confirmed by all the research cited, it was felt that mental health specialists should be warned about the potential need to screen for SRED in patients presenting these factors.

#### 3.1.1 The drug-induced SRED

Regarding the influence of various risk factors over the onset of SRED, there is considerable evidence supporting the impact of certain drugs, such as the Z-drugs and several benzodiazepines, all of which share the activation of type A gamma-aminobutyric acid (GABA) receptors. Although the Z-drugs (zolpidem, zaleplon, zopiclone, eszopiclone) target more specifically α-1 subunits of the GABA-A receptors due to their chemical structure (they are imidazopyridines), unlike benzodiazepines or barbiturates, these agents share common pharmacological effects with other GABA-A receptor agonists, the risk of abuse and physiological dependence being included (111, 112). Zolpidem, several second and third-generation antipsychotic agents (e.g., olanzapine, quetiapine), and antidepressants (e.g., serotonin selective reuptake inhibitors-SSRIs, bupropion) have been associated with sleepwalking or SRED (42–44). Hypnosedative drugs have been associated with various complex sleep behaviors, such as sleep-driving, sleep cooking, SRED, sleep talking, sexsomnia, etc. (45). A review that included ten case reports (N=17 patients) on such complex sleep behaviors induced by non-benzodiazepine receptor agonists concluded that zolpidem (N=15), and far more rarely zopiclone (N=1) and zaleplon (N=1) are triggers for sleep eating, sleepwalking with object manipulation, sleep driving/conversation/sex/shopping (45).

In a study exploring the World Health Organisation (WHO) pharmacovigilance database (VigiBase^®^) (113), out of the 676 drug-associated SRED cases, the most frequent reports involved zolpidem (~36%), sodium oxybate (~27%) and quetiapine (~14%) (46). Another study with a similar methodology questioned the WHO pharmacovigilance database (more than 18 million adverse events reported) and a total number of 508 SRED cases were found to present a possible involvement of these drugs, out of the 5784 reports of SRED and somnambulism (47).

In a cross-sectional study (N=1318 patients taking hypnotics), SRED was reported in 8.4% of the cases, especially in young individuals presenting higher Pittsburg Sleep Quality Index (PSQI) scores, who were receiving higher doses of diazepam-equivalent doses of hypnotics (48). Subjective adverse effects of hypnotics were present in a significantly higher proportion in patients with SRED; also, taking two or more types of antipsychotics was significantly associated with SRED-type experiences (48).

In a retrospective study, out of the 125 patients enrolled, diagnosed with depressive disorders, anxiety disorders, adjustment disorders, somatoform disorders, or sleep disorders, who were using hypnosedatives, 19 (~15%) presented complex sleep-related behaviors (49). All patients with these behaviors were receiving zolpidem, were younger, more frequently female, took higher doses of Z-drug (over 10 mg/day), and tended not to go to bed immediately after the drug’s ingestion (49).

According to a literature review (n=40 case reports), SRED was associated with zolpidem administration, especially when doses placed at the higher end or outside the therapeutic interval (≥10 mg/day) were administered (50). The relationship between the duration of zolpidem use and the onset of SRED may vary significantly, from one single dose to nine years. The causality of zolpidem administration in the development of SRED was supported by the resolution of this condition in all patients who discontinued the administration of the Z-drug (50). The effect of zolpidem on increasing phase 3 NREM sleep may be responsible for the onset of sleepwalking or SRED In a case series of SRED (N=8 patients), zolpidem (10–12.5 mg/day), administered for sleep disorders, was considered the trigger of these nocturnal eating behaviors (1–8 episodes/night, followed by complete amnesia, and their onset was reported 39.8 days after the treatment initiation) (51).

A video report of SRED explored the clinical characteristics of a 53-year-old Caucasian woman with a history of blood hypertension and dyslipidemia who received 10 mg of zolpidem for five years (52). From the onset of the SRED behaviors up to the initial visit for clinical evaluation, the patient’s body weight increased by 6 kg during one year. The discontinuation of zolpidem led to SRED disappearance (52).

Sleepwalking, SRED, and sleep-driving were reported as caused by zolpidem use in a 51-year-old African-American woman with a history of mild obstructive sleep apnea (OSA), hypertension, hyperlipidemia, and depression (53). Zolpidem immediate release (IR) was initiated for insomnia, and after a few weeks, she presented the onset of the previously mentioned sleep-related behaviors. Gradual discontinuation of zolpidem was associated with the immediate ceasing of SRED and related disorders. Two 18-F-fluorodeoxyglucose positron emission tomography (18-F-FDG-PET) recordings were collected two months after zolpidem discontinuation and 1h after a single-dose rechallenge of zolpidem, with no significant differences between them (53).

In a case series, five patients were monitored for 11 months for episodes of nocturnal eating behaviors followed by amnesia that had their onset after zolpidem administration (54). These patients had comorbid RLS (N=5), OSA (N=3), sleepwalking (N=2), or psychophysiological insomnia (N=1) Nocturnal eating behaviors were remitted in all cases once the Z-drug was discontinued (54). In a 45-year-old man who presented symptoms of RLS, zolpidem IR (10 mg) was initiated for the control of initial insomnia, the Z-drug being administered twice per week, on average (55). Nocturnal polysomnography after zolpidem discontinuation revealed a reduced N3 stage, 10-minute sleep latency, and 84.3% sleep efficiency. After zolpidem discontinuation, SRED remitted and did not re-occur at the 6-month follow-up (55).

Zolpidem controlled-release (CR) (6.25 mg/day) was administered for insomnia in a 46-year-old woman with a history of depression, hypertension, mild OSA (treated with continuous positive airway pressure, CPAP), and hypothyroidism, and it induced amnestic nocturnal eating behaviors starting three weeks after the initiation of the Z-drug (56). Switching the patient from zolpidem to eszopiclone led to the complete disappearance of nocturnal eating (56).

A 45-year-old man with obesity (BMI=35.85 kg/m^2^) developed, after ten days of treatment with zolpidem IR 10 mg/day for insomnia, sleepwalking and nocturnal eating behaviors (57). This episode was followed by complete amnesia of nocturnal eating. Stopping zolpidem led to the cessation of SRED behaviors (57).

Zolpidem CR (12.5 mg/day) was considered the trigger for sleep-related complex behaviors (i.e., opening windows, walking, going out of his own house, and SRED behaviors) in a 71-year-old Korean male who presented complete amnesia for these episodes (58). This patient was also diagnosed with RLS and OSA, and he had a low baseline serum level of iron (53 μg/dl, laboratory normal range 60–180 μg/dl). Although SRED disappeared once zolpidem was discontinued, RLS symptoms were not impacted; however, these last-mentioned symptoms disappeared after a single i.v. iron injection (58).

Zolpidem was also associated with the induction of SRED in a 49-year-old man, diagnosed with bipolar disorder, ischemic heart disease, arterial hypertension, obesity, and OSA (initially treated with CPAP, but he did not tolerate this intervention) (59). Lamotrigine (200 mg/day), clonazepam (2.5 mg/day), and zolpidem (10 mg/day) were initiated 14 months prior to the SRED diagnosis. The EEG recordings were normal. The nocturnal eating disappeared after zolpidem discontinuation and was absent at 2- and 5-month follow-up visits (59).

A 21-year-old woman, diagnosed with an inattentive type of attention-deficit hyperactivity disorder (ADHD) one year before, received 10 mg of zolpidem at bedtime for insomnia (60). She did not receive any treatment for ADHD and, after zolpidem initiation, her sleep quality improved. Zolpidem was tapered to 5 mg, then discontinued and replaced by clonazepam 0.5 mg/day. After six months, no signs of SRED recurrence could have been detected (60).

Interestingly, in a report of two SRED cases, extended-release (ER) zolpidem was associated with SRED behaviors, but not the IR formulation (61). These patients, a 75-year-old female with RLS, mild OSA, and maintenance-type insomnia, and a 70-year-old white female diagnosed with RLS, complex sleep apnea, and insomnia, received zolpidem ER 12.5 mg at bedtime and developed amnestic SRED behaviors for several consecutive nights. Discontinuing the ER zolpidem and switching on zolpidem IR led to the remission of these episodes, and this beneficial effect was preserved at 6 months and 12 months of follow-up visits (for the first and second patients, respectively) (61).

Although a Z-drug itself, zaleplon is not associated with many reports of SRED behaviors onset. In one such case report, a 49-year-old woman diagnosed with major depression and treated with duloxetine, who did not test positive on a sleep study for any disorders, received zolpidem up to 15 mg/day for insomnia, which determined the onset of SRED behaviors, with a frequency of four episodes/week (62). She was switched on zaleplon 10 mg nightly, titrated up to 20 mg, and the SRED re-appeared after 25 months of treatment, with a frequency of 1–4 episodes/week. After zaleplon was discontinued, no episodes of nocturnal eating appeared during the next six months (62).

Triazolam administration was related to the onset of SRED. For example, in a case report, a 48-year-old Japanese woman who was diagnosed with type 2 diabetes mellitus, hypertension, sleep apnea syndrome, depression, and RLS, presented nocturnal eating episodes with partial or complete amnesia, which improved after the dose of triazolam decreased from 0.25 mg to 0.125 mg (63). The frequency of abnormal nighttime eating behaviors (that included eating inedible items, such as soap bars) decreased, and this dose adjustment also improved the recollection of her memories of these episodes (63). In a case series of 19 adults, triazolam abuse (0.75 mg) was associated with the onset of SRED (33, 64). In this case series, nocturnal eating appeared immediately after triazolam abuse, and the discontinuation of this benzodiazepine led to the disappearance of SRED manifestations (33).

In a 9-year-old male patient with primary clonus dystonia, insomnia, and severe ADHD, initiating clonazepam at 0.5 mg/day led to SRED, which was remitted after benzodiazepine’s discontinuation (65).

Sodium oxybate is the sodium salt of γ-hydroxybutyrate, which acts as an inhibitory neurotransmitter, and its administration was associated with cases of somnambulism and SRED (66). In a 42-year-old man, undergoing treatment with sodium oxybate 4.5 g/night, titrated up to 8 g/day, who presented with narcolepsy-cataplexy and moderate OSA, the onset of complex activities during sleep was reported two weeks after the maximal dose was reached. No EEG abnormalities were observed in this patient. The abnormal nocturnal behaviors disappeared after the dose was decreased to 7 g nightly (66).

Antipsychotics, both first-generation and second-generation, have been associated with the onset of SRED. Regarding the first category of antipsychotics, haloperidol determined RLS but also “nocturnal eating/drinking syndrome” (an older term encompassing SRED and NES) in a 51-year-old female patient with schizophrenia (67). According to the polysomnography recordings, low levels of sleep efficacy, periodic leg movement, and a correlation between nocturnal eating behaviors and non-REM sleep were observed (67). From the atypical antipsychotics class, olanzapine was reported as an inducer of SRED in a 52-year-old man diagnosed with type I bipolar disorder and undergoing maintenance treatment with lithium (750 mg/day) (68). Adding olanzapine (10 mg/day) for a hypomanic episode (with a lithium level of 0.6 mEq/l) was followed by sleepwalking and nocturnal eating episodes with complete amnesia. Olanzapine discontinuation led immediately to the remission of these SRED behaviors (68).

Aripiprazole may be associated with the onset of SRED behaviors, according to a disproportionality analysis that calculated a 3.6% incidence out of all drug-induced SRED cases (N=676) (46). In a case report, a 41-year-old Japanese man diagnosed with depression and undergoing treatment with sertraline (100 mg/day) received aripiprazole (3 mg) as add-on therapy (69). After three weeks of combined treatment, episodes of nighttime eating followed by complete amnesia were reported by the patient’s mother. Reducing the aripiprazole daily dose to 1.5 mg led to the rapid and complete disappearance of SRED-like behaviors (69).

Quetiapine was the most frequently reported antipsychotic associated with SRED, with over 53% reports in an analysis of the WHO pharmacovigilance database (47). The association between the explored drugs and SRED was significant for second-generation antipsychotics and lithium, but not for first-generation antipsychotics. Quetiapine was considered the trigger for SRED in a 48-year-old woman diagnosed with rapid-cycling bipolar disorder; after two days of quetiapine, 100 mg at bedtime, somnambulism and nocturnal eating followed by amnesia were observed (70). In another case series, quetiapine-induced sleepwalking and SRED-like behaviors were reported in a 51-year-old African male with obesity and severe sleep apnea (150 mg quetiapine at bedtime, for more than a year, the antipsychotic being recommended for depression), and in a 50-year-old African American woman with obesity and mild sleep apnea (200 mg quetiapine, for more than a year, with the same indication as the first patient) (71). In both cases, SRED behaviors were remitted after the quetiapine’s discontinuation and CPAP therapy. Quetiapine was associated with new-onset SRED in a case series (N=2 patients) when administered in doses of 50–200 mg/day (43).

Risperidone was associated with SRED onset in a 68-year-old man with a psychotic disorder due to vascular dementia (72). When the dose of antipsychotic was increased to 2 mg/day, in order to control hallucinations, delusions, and disorganized behaviors that severely impacted his daily functioning, nocturnal eating behaviors appeared, followed by complete amnesia. These eating behaviors persisted for two months, during which the patient received 2 mg/day of risperidone, and disappeared rapidly after the dose was decreased to 1 mg/day (72). In another case report, risperidone 1 mg/day at bedtime was initiated in a 16-year-old girl for aggressive behaviors such as stealing, property damage, and verbal outbursts; after three days of treatment, approximately two hours after sleep onset, she started to present nocturnal eating behaviors; these episodes appeared 2–3 times/week and led to a significant body weight gain (73). Decreased sleep efficiency was observed on polysomnography and the eating episodes disappeared only after risperidone was stopped; during the 1-month and 6-month follow-up visits, she was free of SRED behaviors (73).

Ziprasidone was considered the trigger of SRED episodes in a 28-year-old white male diagnosed with schizoaffective disorder (74). After the dose of ziprasidone was stabilized at 120 mg/day, the patient started to present sleepwalking and SRED for most nights, during the first part of the sleep, followed by complete amnesia in the morning. The treating physician discontinued the nighttime dose, and preserved only 40 mg in the morning, which led to the disappearance of SRED; a re-challenge of 120 mg/day ziprasidone was initiated later, but the nocturnal eating reappeared (74).

From the category of antidepressants, mirtazapine (an inhibitor of the central presynaptic α2-adrenergic receptors) was associated with the onset of SRED in a 24-year-old female patient admitted for depression (75). Episodes of nocturnal binge eating with complete amnesia were also reported by this patient while undergoing treatment with fluoxetine (40 mg/day), trazodone (75 mg/day), and zolpidem (10 mg/day), and an increase of 20 kg in 6 months was also reported; switching her on mirtazapine (30 mg/day) and clonazepam (0.25 mg/day) led to the improvement of depressive symptoms and remission of nighttime eating episodes, but after two weeks of treatment these episodes reappeared, with an onset at 1–2 hours after going to bed. Reducing the dose of mirtazapine to 15 mg/day was not associated with improvements in night eating behaviors, but discontinuation of the antidepressant led to the disappearance of SRED episodes (75). A 19-year-old woman diagnosed with SRED and anxiety, depressed mood, and suicidal ideation received treatment with low-dose mirtazapine and was gradually titrated up to 30 mg when she began to develop episodes of nocturnal eating with partial amnesia (76). During these episodes, she ate multiple packs of crisps, boxes of biscuits, and other food left near her bed, but also presented abdominal pain and vomiting during the night, and a 4 kg increase in her body weight These episodes persisted as long as she received 30 mg of mirtazapine at bedtime but remitted when the dose was lowered to 15 mg (76).

Antidepressants from the serotonin and norepinephrine reuptake inhibitors class (SNRIs), mainly duloxetine and venlafaxine, were also associated with SRED reports (2.7%, and 2.1%, respectively), in an extensive analysis of the WHO pharmacovigilance database (46).

In a 33-year-old white man presenting with nicotine use disorder (with a Fagerström score of 8), bupropion sustained-release (SR) 300 mg/day combined with motivational counseling was considered the most appropriate therapeutic intervention (77). After 17 days of treatment, nocturnal eating episodes (with high caloric intake), sleepwalking, and telephone use during sleep were reported, with partial or complete amnesia. After five weeks of treatment with bupropion, this antidepressant was discontinued and the nocturnal eating and somnambulism episodes disappeared rapidly (77).

Amitriptyline (200 mg/day) caused night-eating behaviors for five years in a 33-year-old woman, even from the beginning of its administration for the control of migraine symptoms; within one week of amitriptyline discontinuation, the night-eating episodes remitted and did not reappear up to the 18-month follow-up (64). This patient also presented sleepwalking with complex and violent, non-appetitive episodes, and the RLS diagnosis was supported by the polysomnographic recordings. Amitriptyline was not the only tricyclic antidepressant involved in the onset of SRED-like episodes, as reported by the same study. An unspecified tricyclic agent probably caused SRED behaviors in a 58-year-old woman diagnosed with depression, without a history of sleepwalking or RLS, and without any signs of recurrence after two years of drug discontinuation (64).

There are also reports that could not significantly correlate the use of dopaminergic or hypnotic drugs for RLS with the presence of SRED, but support a trend for the first category of drugs (67% vs. 52%, p=0.20) (78). The administration of dopamine agonists (l-dopa/carbidopa, bromocriptine) was, however, involved in the onset of abnormal eating behaviors in patients with RLS and Parkinson’s disease (64, 79). Although these reports do not specifically mention the existence of SRED, at least some of the cases could be related to this pathology.

Psychostimulants may trigger SRED-like behaviors, and (dex)amphetamine, lisdexamphetamine, armodafinil, methylphenidate, modafinil, and phentermine were associated with such abnormal eating behaviors in 0.4–1.5% of the drug-induced SRED cases (46).

#### 3.1.2 Psychiatric and organic disorders as potential risk factors for SRED

Regarding the correlation between psychiatric disorders or symptoms and SRED, outpatients with daytime eating disorders and SRED were more depressed and presented a trend towards more dissociation (according to the Beck Depression Inventory, BDI and Dissociation Events Scale, DES scores, respectively) than patients without nocturnal eating (80). Due to the design of the cited case-control study, no temporal relationship could be established between SRED and depression/dissociation; this report just signals their co-occurrence.

Both *daytime eating disorders, NES, and other sleep disorders* were reported in patients with SRED. In a case series (N=19 patients), alcohol and other substance abuse were detected in 21% of these patients (33). In a case series with 19 adults (mean age 40 years), SRED was associated with OSA, more specifically eating during apnea-induced confusional arousals, periodic limb movement + OSA, familial sleepwalking, sleepwalking + periodic limb movement disorder, sleepwalking + irregular sleep/wake pattern disorder, familial RLS, anorexia nervosa with nocturnal bulimia, and amitriptyline treatment for migraines (64).

Patients presenting other sleep disorders are more predisposed to the onset of SRED (44). SRED and nocturnal eating behaviors are frequently detected in relation to RLS, possibly due to the mistreatment with sedative agents (32). A review found that OSA, depression, and RLS were the most frequently reported disorders in these patients’ medical history, and new-onset SRED was present even in patients well-treated for these comorbidities (50).

A survey that took place in a sleep disorder center (N=53 patients presenting RLS) showed that 66% of the responders had frequent night-eating behaviors, and 45% had SRED (81). According to a case-control study (N=100 patients with RLS and 100 matched controls), SRED was more frequently associated with RLS than with the control condition (33% vs. 1%) (78). Also, patients with both SRED and RLS used more medication and had higher scores on the Maudsley Obsessive-Compulsive Inventory (MOCI) than patients presenting RLS without SRED (78).

In a cross-sectional study, nocturnal eating episodes were reported in 31% of the 120 patients diagnosed with RLS, but the difference between SRED and NES was not explored (82). However, these episodes were more frequently detected in RLS patients than in the general population, indicating the need to carefully analyze the comorbidities of patients with RLS (82).

OSA and SRED can be detected frequently together, although one of them may be incidentally discovered in a patient diagnosed and treated for the other (83). OSA may be caused by weight gain secondary to the SRED, or both conditions can be caused by a single incident (such as a head trauma with nasal and mandibular injury, according to a case report) OSA may precipitate the onset of SRED because it interrupts sleep (83).

Narcolepsy and OSA also may be predisposing factors for the SRED onset (84). Patients presenting narcolepsy with cataplexy had a high prevalence of SRED (32%) in a cross-sectional study that compared 65 consecutive adult patients with narcolepsy and a similar number of healthy controls (85). Patients with narcolepsy and cataplexy had more frequently an eating-related pathological profile on Eating Disorder Inventory-2 (EDI-2) (80% vs. 46% in healthy controls). Also, a higher prevalence of depressed mood was detected in these patients, according to the BDI scores, vs. patients without SRED. Patients with narcolepsy and SRED were more frequently women, presented higher scores on “bulimic” and “social insecurity” factors on the EDI-2, had higher obsessive-compulsiveness on the Maudsley Obsessive-Compulsive Inventory (MOCI), and were more depressed according to the BDI scores, compared to patients with narcolepsy but without SRED (85).

Daytime eating disorders were reported in 10% of the SRED patients (64). In a previously cited, 5-year study (N=19 participants), SRED was also often accompanied by other dysfunctional behaviors during the night, with only two patients presenting an eating disorder (anorexia nervosa), and almost 50% having another psychiatric diagnosis, such as mood and anxiety disorders (33). A personal history of eating problems in childhood and higher current anorexia scores were reported in patients with SRED vs. healthy controls or patients with sleepwalking (86).

*Parkinson’s disease (PD)* was considered a possible trigger for SRED behaviors. In two patients with early-onset PD, a 28-year-old male and a 37-year-old male, SRED was confirmed by audio-video polysomnography (87). In the first patient, besides SRED, episodes of confusional arousals during N3 sleep were recorded, while in the second case, OSA, NES, and RBD were present as comorbidities. Sleep hygiene education and a decrease of pramipexole daily dose to 1.5 mg, together with the introduction of 150 mg levodopa daily, led to the disappearance of SRED in the first patient, while in the second patient, the correct treatment of OSA with CPAP and sleep hygiene education were sufficient to cancel the nocturnal eating behaviors (87). A 56-year-old woman, with a two-year history of PD, was also diagnosed with hypothyroidism (undergoing treatment with levothyroxine), depression (treated with nortriptyline), OSA (but could not tolerate CPAP treatment), sleepwalking, REM sleep parasomnia, and bruxism; SRED was also detected, based on her husband’s reports, without any daytime eating disorder (88). SRED in patients with PD has been conceptualized as a comorbidity or an adverse effect of dopaminergic activators. However, pramipexole, which is a dopaminergic activator, was associated with favorable results in patients with SRED (91).

#### 3.1.3 Other factors associated with risk of SRED onset

Work-related stress, disturbance of the circadian rhythm due to professional tasks, or insufficient time allocated to sleep were reported as risk factors in a case series (84). Acute stress derived from worries about the safety of family members or relationship problems triggered SRED (64).

### 3.2 Comorbidities of SRED

According to the current evidence in the literature, the most frequently reported comorbid conditions in patients with SRED are insomnia (58.8%), RLS (47%), sleep-disordered breathing (26%), various primary psychiatric disorders (38%), and overweight or obesity (41%) (114). As previously mentioned, outpatients with SRED and daytime eating disorders had more symptoms of other sleep disorders and higher levels of dissociation and depression than patients without SRED (80). Although the temporal sequence is unclear, because the cited study was focused on measuring the prevalence and comorbidity of nocturnal eating, not on causation, a comprehensive screening of patients with SRED for other psychiatric disorders is granted based on the available data. Also, patients with a daytime eating disorder are more likely to be diagnosed with SRED, especially hospitalized individuals, than other clinical populations (e.g., obese patients, depressed subjects) or randomly selected controls (i.e., college students) (80).

Within the SRED-diagnosed patients, the history of a daytime eating disorder is more likely, and 35% of such patients enrolled in a case series (N=23) admitted a lifetime eating disorder diagnosis (115). In another case series, 67% of the 15 patients with SRED also had bulimia nevosa, and 33% had anorexia nervosa (80).

RLS and nocturnal eating have been reported frequently as appearing together in clinical settings, but the currently available evidence could not support a causal relationship. A „restless nocturnal eating” syndrome was suggested by several authors, as a synthesis of the two clinical entities, because restlessness and nocturnal eating arise, reach their peak, and then decrease together (116).

Different parasomnias, consisting of REM sleep behavior disorders and NREMSADs, may occur in the same individual, representing the parasomnia overlap disorder (POD) (117). A 42-year-old male was diagnosed with severe OSA, sleepwalking, SRED, and sexsomnia; video polysomnography confirmed arousal from N3 sleep and an overall increase of muscle tone during REM sleep; nasal CPAP improved sleepwalking and SRED, but sexsomnia was improved only after clonazepam 0.5 mg at bedtime was administered 7 (117).

A high rate of SRED comorbidity was present in patients with narcolepsy, according to a study that included 710 patients with type 1 or type 2 narcolepsy, and idiopathic hypersomnia (118). SRED was more frequent in patients with type 1 narcolepsy (7.9%) and was associated with disrupted nighttime sleep (OR=3.9) and nocturnal eating with full awareness (OR=6.9) (118).

Weight gain and obesity may appear due to frequent nocturnal eating, and other daytime eating disorders have been described in patients with SRED (80). Significant weight gain was reported and associated with SRED, even in patients who were on a diet and regularly exercising (84). Also, being overweight was a variable detected in 44% of 38 patients within the SRED series (33, 64).

SRED and DOAs have a certain relationship, supported by the high frequency of present or personal history of sleepwalking in patients with nocturnal eating, the onset of dysfunctional eating in the first half of the night, and possible arousals from the SWS in these patients (119). Also, RLS, periodic limb movements and OSA, as well as recurrent chewing and swallowing movements during sleep, have been reported in patients with SRED who associated lack of control on the eating behaviors during the night (e.g., the ingestion of unpalatable or toxic items, like cigarettes, dishwashing liquid, or animal food) (119). All these arguments support the existence of a common pathophysiological background between DOAs and SRED, or at least a common vulnerability terrain.

The pre-bariatric surgery evaluation is recommended to include an assessment of SRED and NES. In a case report, a 38-year-old woman complained, on her sixth day after sleeve gastrectomy, about a nocturnal eating episode followed by complete amnesia (88). She woke up in the morning with the taste of food in her mouth and she found crumbs on her pillow, although she was on a strict post-surgery liquid diet She admitted she had previous SRED episodes since the age of 20, but did not seek medical help, and such behaviors were not included in the pre-surgical interview Her body mass index (BMI) was 48.9 kg/m^2^ prior to the surgical intervention, and behaviors corresponding to NES and binge eating disorder (BED) were identified, once she was referred (post-surgery) to a specialist in eating disorders. She also reported the existence of a positive familial history, with her two brothers being diagnosed with obesity and treated with bariatric surgery, and they also presented episodes of nocturnal eating with full awareness (mean frequency of three episodes/week) (88).

Diabetes mellitus, obesity, and hypercholesterolemia have been reported in patients presenting with SRED, most likely as consequences of high-calorie ingestion during the night (53, 63, 120, 121).

### 3.3 Pathogenesis of SRED

This disorder may be idiopathic or it can be associated with other sleep disorders. For example, SRED has been reported in patients with RLS, OSA syndrome, and other clinical conditions (13). Also, a common pathophysiological background has been suggested for SRED and NES (121).

According to a systematic review (n=15 papers), SRED did not occur during the deep sleep phase, i.e., the N3 stage, and the explored studies could not find significant abnormalities of the polysomnographic parameters (120). Still, in other reports, a dissociated arousal in the N3 stage of sleep has also been observed in patients with SRED and was considered a vulnerability feature for parasomnias (122).

Based on these results, the utility of polysomnography in SRED is not clear, although this investigation may be helpful in differentiating SRED from other eating disorders (120). These conclusions have been based on data evaluated by the authors of the review as presenting a moderate and high risk of bias (120). Still, in a 29-year-old man, SRED had a history of six years, with nocturnal eating episodes appearing up to five/night (8–16 minutes each), and partial amnesia (92). The polysomnography recordings indicate the arising of SRED episodes from N2 NREM sleep, with maintenance of this sleep stage throughout the entire episode, or with wakeful EEG, but without epileptiform activity (92).

In a study that enrolled 23 patients presenting SRED (83% female), the polysomnographic recordings showed the presence of somnambulism in almost 50% of the cases (115). Also, 35% of these patients had a lifetime history of eating disorders. More than 90% of the patients with SRED reported they were „half-awake, half-asleep” during the eating episodes, with significant or partial amnesia of the events. Most of the patients had an onset of their SRED during adolescence and a chronic evolution, with a history of 15.8+/-11.2 years (115). In conclusion, SRED combines features of somnambulism and eating disorders.

A study (N=35 drug-free patients with nocturnal eating) used video polysomnographic recordings in a sleep laboratory to detect the neural signatures of the SRED or NES (123). The vast majority of the patients presenting nocturnal eating in lab settings were fully awake during their episodes, and the onset of the dysfunctional behavior was observed after non-REM sleep; only one patient had the onset of this behavior during awakening from REM sleep (84). All patients presented alpha activity on their EEG and no dissociated features during their nocturnal eating These patients had a history of sleepwalking (N=1), somniloquy (N=5), RLS (N=8), and periodic limb movements during sleep (N=4) (84). During lab conditions, periodic limb movements were present in 22 patients, RLS dyskinesias in 5 patients, and recurrent chewing and swallowing movements during sleep in 29 patients (123).

The relation between glucose metabolism and SRED was explored from a pathophysiological perspective. A decrease of this metabolism at the cortical level during physiological sleep was reported, but in zolpidem-induced sleep, this correlation was not supported by evidence (53). This observation suggests that some instinctive behavioral patterns related to survival may be abnormally activated during the administration of zolpidem, a drug frequently invoked as an SRED trigger (53, 59). Another theory suggests that SRED induced by zolpidem is a consequence of this drug’s inhibitory activity on the serotonin neurotransmission at the hypothalamic ventrolateral nucleus, which controls the food appetite, or at the level of raphe nuclei, which changes their activity depending on the feeding status (53). On video-polysomnography, chewing and swallowing movements during N2 sleep have been reported in patients with SRED (122, 123). It was hypothesized these behaviors may represent an anticipation of the reward, represented by eating, similar to the results of translational research (122).

The enhancement of GABA-ergic activity especially at the α-1 subunits of GABA-A receptors, determined by Z-drugs, increases the risk of complex sleep behaviors onset (45). The amnesia that follows such behaviors is also a possible consequence of GABA-ergic enhancement, a phenomenon that inhibits the consolidation of short-term memories. Therefore, lowering the dose, discontinuation, or switching to another drug are recommended in patients treated with Z-drugs who develop such complex sleep behaviors (45).

Serotonin (via 5HT2 receptors) may have an inhibitory effect in controlling SWS (73). Atypical antipsychotics may increase SWS due to the reduction of serotonergic transmission A decrease in the dopaminergic neurotransmission is also a possible cause of SRED that may explain the association of certain antipsychotic drug use with new onset SRED (73).

The decrease in dopaminergic and/or serotonergic activity may be a factor that leads to SRED (89). The co-occurrence of RLS and SRED supports the involvement of dopaminergic dysfunction in these patients (75).

High levels of novelty seeking, exploratory excitability, and increased reward sensitivity were reported in a case series (N=2) of patients with SRED (122). Because compulsive eating disorders have been associated with a more intense mesolimbic dopaminergic sensitivity, it was hypothesized that the activation of the reward system during sleep makes the individual more vulnerable toward the onset of nocturnal overeating, especially in the presence of high novelty seeking and reward sensitivity. Motor disinhibition during nighttime, typically met in parasomnias, combined with the arousal of regions within the reward system, can explain the onset of SRED (122).

There is a distinct possibility that an internally generated stimulus can determine partial arousal and, if this arousal occurs during non-REM sleep, in an individual with a certain predisposition, may induce a nocturnal eating episode (72, 115). Hypoglycemia or unconscious emotional stimuli may be triggers for eating in inappropriate conditions (72, 124).

Genetic factors may contribute to the vulnerability to „disorders of arousal”, but evidence to support this hypothesis is insufficient (14). In a case report, SRED was detected in the index case and her fraternal twin sister and father, supporting the claim of a genetic vulnerability (125). The index case did not respond to clonazepam, but responded to an OTC for cold, containing pseudoephedrine hydrochloride, dextromethorphan hydrobromide, doxylamine succinate, and ethyl alcohol (125).

### 3.4 Differential diagnosis

An important differential diagnosis is represented by the night-eating syndrome (NES), which refers to patients who are unaware of the time and quantity of food they eat (32). NES is considered by several authors a non-motor group of clinical manifestations related to RLS (32). In the seminal work by Stunkard et al. (30), NES was reported as an eating pattern defined by nocturnal hyperphagia („the consumption of large amounts of food during the evening and night… (of) at least a quarter of (their) total calories for the day during the period following the evening meal”), insomnia („sleeplessness, at least until midnight more than half of the time”), and morning anorexia („negligible food intake at breakfast”) in a group of 25 individuals with obesity, who were compared to 38 individuals without a history of weight disorder (30). According to the DSM-5TR criteria, NES is included in the category of „Other specified feeding or eating disorder” (9). This is considered progress in the way of achieving an independent nosological status for NES, because in the previous edition, DSM-IV-TR, this disorder was included in the category of „Eating disorders not otherwise specified” (126). According to the DSM-5TR, for diagnosing NES, the following criteria should be present: recurrent episodes of night eating (eating after awakening from sleep or excessive food ingestion after the evening meal), awareness preserved about the eating episodes, the recall of such episodes is possible, there are no external influences in the sleep-wake cycle or changes in local social norms, there is significant distress and/or impairment of functioning, and there is no other medical, substance-related, or psychiatric condition that may better explain these symptoms (12). SRED patients had more clinical manifestations suggesting a sleep disorder, more severe depression, and more intense dissociation than patients with NES, according to a study evaluating the comorbidity in patients with night eating, without focusing on a causality relationship (80). Other authors report higher scores on physical tension, mood, and sleep dysfunctions in relation to NES than in relation to SRED, with no significant differences in age, BMI, or gender distribution (127).

In a clinical comparison that explored the medical records of 30 patients with primary SRED and 10 patients with drug-induced SRED (pharmacological agents with sedative properties, mainly zolpidem or benzodiazepines), a higher mean age of onset (40 vs. 26-year-old), higher rate of total amnesia (75% vs. 32%), lower rate of comorbid NES (0% vs. 63%), and lower rate of sleepwalking history (10% vs. 46.7%) were present in patients with drug-induced SRED (128). The combination of multiple types of sedative agents was observed in all patients with SRED induced by drugs (114). Higher doses of benzodiazepines may be responsible for the onset of SRED. The polysomnography recordings showed in drug-induced SRED a longer sleep-onset latency and a lower duration of deep sleep, possibly related to insomnia or pharmacologic effects of sedatives in these patients (128). Unlike SRED, the existence of drug-induced NES was not demonstrated (59).

During the SRED episodes, unlike during NES, edible and non-edible items can be consumed, and a mixture of these types of items is also possible. For example, in a patient with SRED (unspecified gender or age), easily accessible foods (milk, candies, fruits, or leftovers) were consumed in a disorganized manner, but the tentative consumption of a self-prepared meal consisting of bread and dishwashing liquid was the fact that alerted the family and led to the specialized consultation (129).

There is a controversy in the literature about the differences between NES and SRED, and whether the two disorders are, in fact, a single syndrome or two distinct disorders (130). Although both disorders involve dysfunctional eating behavior during the nighttime, with high-calorie intake and multiple awakenings, higher prevalence in women and possible familial aggregation, there are still several significant differences: the level of consciousness during the eating episodes (full awareness in NES vs. partial/complete lack of awareness in SRED) with consecutive full remembrance of the episodes (in NES) or impaired recollection about them in the following morning (in SRED) (116). One hypothesis states the two entities are, in fact, a single disorder, considering that sleep disorders specialists and eating disorders specialists focus their research on the same dysfunctional eating behavior, but from different perspectives, thus creating the impression of two distinct pathologies. According to this hypothesis, SRED is just an epiphenomenon of sleepwalking, while NES is a type of binge eating disorder with nocturnal manifestations (130).

The differential diagnosis of SRED also involves other conditions characterized by the presence of abnormal eating during nighttime (131). *Kleine-Levin syndrome* (KLS), defined by a fluctuating course and an onset during adolescence, is a rare disease with episodes lasting from one to several weeks, and its core manifestations are hypersomnia, confusion, slowness, amnesia, derealization, and apathy (132). The pathophysiological substrate is supposedly recurrent inflammatory encephalitis, but a genetic component is also suggested by the fact that 5% of cases are familial (132). The confusion with SRED may arise from the hyperphagia observed in patients with KLS because 2/3 of them have episodes of compulsive eating, which can be reported during their sleep (132).

*BED* and episodes of nocturnal eating in patients with *bulimia nervosa* also need to be explored in the context of a differential diagnosis of SRED (131). Recurrent episodes of binge eating are characteristics of BED and bulimia nervosa, but compensatory behaviors targeting weight gain prevention are typical only for bulimia nevosa (12). Because these binge eating episodes may be present not only during daytime, SRED should be included as a potential differential diagnosis. However, while such episodes are accompanied by partial or complete amnesia in patients with SRED, in bulimia nervosa and BED the patients are fully aware of their behavior.

*Dissociative disorders* may involve episodes of eating followed by amnesia, including during nighttime (131). These disorders disrupt the continuity of integrating data into consciousness and impair the functionality of memory, perception, motor control and behavior, therefore creating confusion between SRED and dissociative eating behaviors (12). Still, dissociative disorders are complex, and their symptoms are virtually never limited only to eating behaviors.

The distinction between *non-dysfunctional nocturnal eating* (NDNE) and SRED is important, in order to avoid stigmatization and the tendency to over-pathologize normal behaviors. Non-dysfunctional nocturnal eating is presumed to be a non-pathological variation of SRED or a subtler form of SRED (32). Although a clear definition of NDNE could not be retrieved in the literature, based on the existing studies, it may be considered that in this case, the core criteria of SRED are not met. For example, those criteria referring to partial/complete amnesia of eating episodes, the possibility of ingesting nonedible items, or potentially dangerous behaviors due to automatic behaviors are not met in NDNE (26, 30). The confusion between SRED and NDNE may arise from the nocturnal eating episodes and from the adverse negative effects on one’s own health, like obesity or metabolic syndrome.

### 3.5 Epidemiology

In patients with eating disorders, depression, or obesity, an estimated 4–5% prevalence of SRED was based on epidemiological studies (80). Hospitalized patients with eating disorders have a much higher prevalence of SRED than outpatients with such pathology and the control group (16.7% vs. 8.7% vs. 4.6%) (80).

A majority of women (67.6%) were reported in a study evaluating the demographical characteristics in a group of 23 patients with SRED (114). This gender distribution was confirmed in another study (N=15 patients with SRED), and the onset of the disease was in adulthood (86). In a review of zolpidem-related-SRED cases (N=40), the majority of patients were also females (65%), who used high doses of this drug (10–30 mg in 95% of the situations) (50). According to this review, these patients also had a high percentage of concomitant SSRIs or benzodiazepines use (~57%) (50). Several authors correlated this higher incidence of SRED in women with the higher rate of daytime eating disorders (121).

The mean duration from the first SRED symptoms until the moment of diagnosis was 39+/-13.8 (17–67 years) (114). Other authors considered the average duration from the very first signs up to the first contact with healthcare systems, to be 12–16 years (131). Also, the onset of SRED was reported between adolescence and young age, with most of the patients presenting a chronic course (121). The average age of SRED onset was estimated to be between 22 and 27 years (131).

According to a cross-sectional survey conducted in a Hong Kong outpatient clinic, by interviewing 1235 subjects, a 4% lifetime prevalence of SRED was calculated, compared with 8.5% for sleepwalking, while the 1-year prevalence was 2.4%, and 2.9%, respectively (133). These conditions were associated with depression and various other sleep disorders Sedative antidepressants (tricyclics, trazodone, and mianserin) and nonbenzodiazepine hypnotics (zolpidem and zopiclone) were associated more frequently with sleepwalking, while zolpidem and antidepressants with SRED (133).

Many sources highlighted the chronic evolution of SRED, prior to the treatment initiation (33, 115, 134).

### 3.6 Structured evaluation

The *Inventory of Nocturnal Eating* (INE) was developed to evaluate both night eating behaviors and sleep quality (80). It is a self-administered questionnaire and comprises seven sections, with an unspecified number of items (64). If any nocturnal eating is detected, its frequency and awareness are further explored The interpretation allows differentiation of eating behaviors during sleep and nocturnal eating without loss of consciousness. The communicated inventory’s sensitivity was 0.91 (80).

A questionnaire dedicated to parasomnias and nocturnal behaviors, *Munich Parasomnia Screening* (MUPS), is based on 21 different clinical manifestations (translated into an equivalent number of questions) and it is self-assessed (135). This instrument allows for assessing the lifetime history or frequency of each manifestation on a Likert scale with seven options (121). This instrument’s sensitivity and specificity were >75% (135). Sleep-related eating is evaluated on this questionnaire distinctly from nocturnal eating and confusional arousals. The fact that SRED and all the other parasomnias can be assessed with a single instrument is very helpful for clinicians, although this instrument is not widely used. A Japanese version of MUPS was validated by Komada et al. (136) and the estimated time to complete this questionnaire was calculated to be 8 minutes (range 2–17 minutes) (136).

The *Night Eating Questionnaire* (NEQ) was created to assess the severity of NES, and it can be a useful instrument for differentiating SRED from NES. This questionnaire comprises 14 items, assessed on a 5-point Likert scale, for quantifying behavioral and psychological symptoms of NES and has a Cronbach alpha value of 0.70 (137). A four-factor structure was supported by the analysis, with nocturnal ingestions, evening hyperphagia, morning anorexia, and mood/sleep confirmed as the main dimensions of NES. The original, unpublished version of NEQ contained only nine items, assessed on a 4-point Likert scale, and evaluated morning anorexia, evening hyperphagia, initial insomnia, mid-phase insomnia, nocturnal ingestions, and mood (137).

A self-rating scale (*Paris Arousal Disorders Severity Scale, PADSS*) containing items that describe 17 parasomniac behaviors, assessed for their frequency and consequences, was designed mainly for the evaluation of sleepwalking and sleep terrors but also included descriptors of other NREMSADs (138). This scale has demonstrated high sensitivity, specificity (patients with non-REM vs. REM parasomnias and patients with arousal disorders vs. normal controls), internal consistency, and test-retest reliability, with a cut-off for the total score of 13/14 for non-REM parasomnias. Two components were identified by factor analysis, i.e., „wandering” and „violence/handling. The behaviors emerging from N3 sleep (demonstrated on video-polysomnography) correlated with PADSS total scores, its subscales, and the „violence/handling” factor. PADSS includes an item for SRED (with complete amnesia) that can be rated as „never”, „sometimes”, or „often”, with a self-estimated frequency of the eating episodes over the last year (from none to two or more episodes per night), and for the negative functional/emotional consequences of these behaviors (138).

None of these three instruments can be considered specific for evaluating SRED, but they can be useful for screening purposes (i.e., detecting nocturnal eating behaviors) as a preliminary step to a more detailed evaluation. Also, NEQ may be used to differentiate SRED from NES by providing a severity score for the last diagnosis, and PADSS may distinguish between non-REM and REM parasomnias.

### 3.7 Treatment

The treatment of DOAs is still largely based on the prevention and removal of precipitating factors as well as on clinicians’ personal experience with similar cases (14). In patients where the onset of SRED can be related to the presence of sleepwalking, periodic limb movements, substance use disorders, chronic autoimmune hepatitis, narcolepsy, encephalitis, or acute stress reaction, these conditions should be adequately treated, as an essential stage of the SRED management (33). In patients with sleepwalking, almost 73% had their nocturnal eating and other sleepwalking behaviors suppressed by the administration of clonazepam and/or bromocriptine treatment. Patients with periodic limb movements and SRED responded favorably to combinations of carbidopa/l-dopa, codeine and clonazepam (33). CPAP may be a useful method to approach non-pharmacologically patients with SRED and associated OSA (89), but evidence-based recommendations for the utility of such a therapy are sparse (64, 90). In two reported cases of SRED and OSA dual diagnosis, CPAP succeeded in controlling nocturnal eating behaviors (90). However, efficient treatment of the primary sleep disorder can lead to remission of nocturnal eating behaviors in a significant proportion of cases, therefore any therapy targeting the primary sleep disorder is granted (33, 89).

According to a 2019 literature review, none of the explored treatment options for NES or SRED have proven their long-term efficacy in good-quality studies (93). Lack of information about the existence of SRED among clinicians and a possible under-reporting of this condition by patients may be plausible causes for the lack of evidence-based therapeutic recommendations (93).

However, based on very limited data, first-line treatment for SRED includes SSRIs, with topiramate or clonazepam as alternative options (13). Clonazepam 1 mg at bedtime decreased the nocturnal eating behaviors in a 48-year-old Japanese woman, although her sleepwalking remained (63). Adding pramipexole 0.125 mg to clonazepam improved SRED, RLS, and sleepwalking manifestations (63). Also, clonazepam treatment completely eliminated the episodes of RLS and nocturnal eating in a 51-year-old female patient with schizophrenia (67).

The combination of dopaminergic and opioid agents has also been described as a treatment for SRED, in a series of seven cases (94). Occasional use of sedatives was allowed in these patients, although no associated sleep disorders were detected (94). Dopaminergic agents (carbidopa/l-dopa, bromocriptine) +/- codeine and clonazepam led to favorable results in cases of SRED associated with sleepwalking or periodic limb movement in a series of 19 adults (64).

In a 35-year-old male with a dual diagnosis of obesity and SRED, the initiation of phentermine and topiramate ER led to favorable results after five months of combined treatment, with 5% weight loss and remission of dysfunctional eating behaviors (95). However, after the patient’s self-initiated treatment discontinuation, the SRED symptoms reappeared (95). Topiramate (flexible-dose up to 300 mg) was explored in a 34-patient randomized clinical trial, which enrolled participants with SRED (mean duration of their disorder 13.7 years) (96). After 13 weeks, the primary outcome (percentage of nights with eating behavior) was significantly improved by topiramate vs. placebo and baseline values. More responders to topiramate, as assessed by the Clinical Global Impression-Improvement (CGI-I) scale, than to placebo, were observed, and the level of wakefulness and memory of episodes predicted the favorable therapeutic response for nighttime eating at baseline. Also, the body weight decreased more during topiramate treatment vs. placebo, while paresthesias and cognitive dysfunction were the most frequently reported adverse events (96).

In yet another report, 12 out of the 17 enrolled patients diagnosed with SRED who received topiramate had a favorable outcome and tolerated this drug well over 1.8 years (97). In a case series, two patients diagnosed with SRED and two with NES, with a history of non-responsivity to multiple trials of pharmaco- and psychotherapy, received open-label, naturalistic, treatment with topiramate at bedtime (98). One patient with SRED had a marked response, and one had only a moderate response, based on clinical outcomes The benefits of topiramate treatment were still observed after 8.5 months (98). Also, in a 45-year-old woman diagnosed with sleepwalking, SRED, who also presented sleep-related smoking (none of these confirmed on video polysomnography) and had a history of mild OSA, topiramate (25 mg/day initially, titrated up to 100 mg/day, at bedtime) led to complete resolution of all three pathological nocturnal behaviors; these positive results persisted at 10-month follow-up (99).

A retrospective chart review explored the effects of topiramate in 30 patients with SRED, using as an outcome the variations of Clinical Global Impressions of Improvement (CGI-I); after an 11.6 months mean duration of treatment with topiramate (mean dose 135+/-61.6 mg daily), 68% of the patients were considered responders (CGI-I scores „very much” or „much” improved) (100). The tolerability of topiramate in this study was not very good, and adverse events were reported by 84% of these patients, while more than 40% discontinued the treatment after a mean duration of 12.4 months (100).

Topiramate was efficient in a 28-year-old man who suffered from SRED and sleepwalking with daily episodes for ten years (101). He was overweight and had a personal and familial history of other sleep disorders. After the failure of clonazepam (2 mg/day) and fluoxetine (20 mg/day), topiramate up to 50 mg/day was initiated and led to the complete disappearance of SRED manifestations. Although isolated episodes of sleepwalking remained, the treatment was well tolerated, and after two years, there was no recurrence of nocturnal eating (101).

Sertraline (low dose, 25 mg daily) taken at bedtime induced complete and prolonged (up to 9–17 months) remission of SRED symptoms in two patients (males, 36 and 37-year-old) who had this disorder for 3 and 7 years, respectively (84). SSRIs (fluvoxamine and paroxetine) were efficient in a 4-case study with SRED-diagnosed patients (102). Fluoxetine was efficient in 2 out of 3 patients in a case series of SRED (N=19) (64).

In a case report, agomelatine controlled the sleep-related eating symptoms in a 54-year-old woman diagnosed with depression, panic disorder, SRED, and sleep apnea (103). After cessation of agomelatine treatment, the SRED symptoms reappeared, but they were remitted again after reinitiating the melatoninergic antidepressant.

When SRED is connected to RLS, dopamine agonists (e.g., pramipexole) could be recommended, and sleepwalking-related SRED may benefit from low doses of clonazepam (13). Pramipexol (0.18–0.36 mg) was evaluated in a placebo-controlled study with 11 consecutive patients diagnosed with SRED who did not present daytime comorbid eating disorders (91). After two weeks of treatment, the median night duration of abnormal behavior decreased, and the number of nights with restorative sleep during each week significantly improved. Also, the tolerability of this D3-agonist was good, and no withdrawal from the study was recorded (91).

A combination of bupropion, l-dopa, and trazodone was associated with good results in a case series (N=2 patients with SRED, monitored with polysomnography), in individuals with a personal history of substance use disorders (alcohol, cocaine +/- tobacco) but without other comorbid sleep disorders (104). These two received carbidopa/l-dopa monotherapy without significant effect, but full control of nocturnal eating and nocturnal awakenings was obtained after the addition of bupropion and trazodone. Also, in one case, a significant weight loss was reported and maintained at a one-year follow-up (-6 kg to baseline) (104).

Replacement of benzodiazepines with other, non-GABA-ergic pharmacological agents, can be beneficial in decreasing and eliminating the occurrence of SRED episodes. For example, suvorexant, a selective dual antagonist of the type 1 and 2 orexin receptors (OX1 and OX2), demonstrated efficacy in a 25-year-old woman diagnosed with depression, who presented episodes of night eating with partial or complete amnesia and sleepwalking for one year (105). This patient was diagnosed with SRED based on polysomnography, which detected an awakening from the N2 stage sleep from eating. She was recommended to discontinue brotizolam (0.25 mg) and was switched on to suvorexant (10 mg), which led to a complete disappearance of SRED. During the 2-year monitoring period, she did not present any new SRED manifestations (105).

A retrospective study (N=49 patients) reported on the efficacy of ramelteon as an add-on to the ongoing benzodiazepine treatment, followed by dose reduction of benzodiazepine, as a strategy for patients with SRED (N=45), NES (N=41) or both (N=37) (106). The introduction of ramelteon (4–8 mg/day) led to a mean decrease in nocturnal eating behavior per week from 5.3 to 3.2, and 43% of these patients were responders. Only five patients reported adverse events, consisting of mild daytime somnolence. Also, the mean benzodiazepine and Z-drugs dose (brotizolam, clonazepam, eszopiclone, etizolam, flunitrazepam, loflazepate, triazolam, zolpidem, and zopiclone) decreased significantly from baseline to the post-ramelteon phase (i.e., mean duration of 11.7 months of ramelteon treatment), but only in the responders’ group (106).

A regular follow-up is recommended for all patients with SRED, at least 2–3 times per year, using clinical evaluations and structured interviews to monitor the treatment’s efficacy and its adverse events (13).

Psychotherapy was also tried for patients with parasomnias, SRED cases included. In a study with 36 patients, out of which two were diagnosed with SRED, received one or two hypnotherapy sessions, followed by regular visits up to 5 years (107). Although the overall rate of response was good, with more than 45% at the 1-month visit and more than 40% at the 5-year follow-up free of symptoms or much improved, SRED cases were much improved only in 50% of the cases after 5 years (107). However, due to the small number of patients with SRED, the significance of this percentage is difficult to assess. In a case series of parasomnias, a 38-year-old female with sleepwalking and nocturnal binge-eating episodes reported amnesia for nocturnal eating behaviors; these sleep-eating episodes occurred 2–3 times/week (108). Two sessions of hypnotherapy included suggestions about waking up, feeling calm and safe, and going back to bed when her feet touched the floor or opening the fridge door. The episodes of sleepwalking/SRED significantly decreased (only one episode during a month was reported) (108).

The efficacy of cognitive-behavioral therapy (CBT) for SRED was not systematically assessed in clinical trials. However, there are studies that enrolled patients with SRED in parasomnias-focused CBT that may appear promising (109, 110). CBT-NREMP (CBT for non-REM parasomnias) is a new type of therapy, group-based, focused on decreasing parasomnia severity during a limited number of sessions (109). CBT-NREMP is a development of CBT for insomnia (CBT-I) approach that includes psychoeducation on non-REM parasomnias, sleep hygiene, sleep rescheduling, stimulus control, and specific body-based and relaxation interventions (109). In such a study, 46 patients with NREMSAD (out of which one presented SRED) received a 5-session group-based CBT-NREMP program, and a significant reduction in the outcomes (clinical measures of NREMSAD, insomnia, and anxiety and depression) was observed (109, 110). However, due to the very small number of patients with SRED in this study’s group, it is difficult to extract definitive conclusions on this specific pathology. According to a systematic review of CBT for parasomnias, this type of psychotherapy is ineffective when compared with pharmacotherapy, but the authors acknowledge there is no direct comparison between the two types of interventions in SRED in clinical trials, and their observation is based on a single report (64, 110).

Offering limited access to food before going to bed was associated with modest benefits in a 29-year-old man with a history of 6 years of SRED (92). Another report to support this recommendation refers to a 34-year-old white man, who presented SRED behaviors with a frequency of 2–3 episodes/night, followed by complete amnesia, with the onset of SRED after a motorcycle accident (83). After an incarceration of 1.5 years, he lost 36 kg due to the inaccessibility to food during nighttime. After his release from prison, he gained 40 kg, and SRED episodes were again witnessed by his wife (83).

## 4 Discussion

Various pharmacological agents have been associated with an increased risk of SRED, but the Z-drugs and certain benzodiazepines benefit from the strongest evidence (33, 42–65, 113). Based on primary and secondary reports, the involvement of zolpidem, especially IR, and to a lesser extent zaleplon, triazolam, and clonazepam in the onset of SRED is confirmed. The dysfunctional eating behaviors induced by these agents are reversible if they are discontinued, or if a switch to a different sedative agent is conducted. From this perspective, clonazepam has been associated with paradoxical effects, therefore its prescription should be made with caution (65). Even changing the formulation of the drug, from ER to IR can prove beneficial, although this observation is derived only from a case series (61). Sodium oxybate, antipsychotics, antidepressants, dopaminergic agents, and psychostimulants have also been associated with the capacity to trigger SRED onset (66–79). The exact pathophysiological mechanism involved in the triggering of SRED is not known, and the lack of translational data in this regard, due to the difficulty of creating animal models of SRED, hinders the development and validation of a pathophysiological paradigm for this parasomnia.

Psychiatric and organic disorders have been investigated as potential risk factors for SRED, and there are isolated data supporting the possible negative effects of depression, dissociation, substance use disorders, NES, daytime eating disorders, different sleep disorders, and Parkinson’s disease, but the causal relationship is controversial. In the absence of prospective, large-scale, epidemiological studies it is difficult to infer the exact relationship between SRED and other disorders, either mental or organic.

Work-related stress, disturbance of the circadian rhythm due to professional tasks, insufficient time allocated to sleep, and acute stress have all been associated with a risk of SRED onset (64, 84). However, the evidence to support such an association is weak and based only on case series.

Regarding the comorbidities of SRED, the most frequently invoked conditions were insomnia, RLS, sleep-disordered breathing, sleepwalking, primary psychiatric disorders (especially daytime eating disorders), overweight or obesity, diabetes mellitus, and hypercholesterolemia have been supported by evidence (80, 114, 115, 117). Due to the high rate of comorbidity reported in patients with SRED, reaching almost 60%, a detailed screening for organic and psychiatric disorders is required in these patients. Other psychiatric disorders associated with high levels of anxiety or high frequency of insomnia should benefit from more exploration of possible SRED-associated behaviors (126, 139–141). Other pathologies of potential relevance for SRED are those that alter sleep quality, including bruxism, temporomandibular diseases, and chronic pain-associated pathologies (23, 24). Although there is virtually no data on the impact of these disorders on SRED, this topic is worthy of further investigation since they may trigger nocturnal awakenings and significantly impact the quality of sleep. For example, sleep bruxism was associated with 1.28 times and awake bruxism 1.14 times lower quality of sleep (142–144).

The pathogenesis of SRED is still in its early research phase, but two forms have been described- the idiopathic and the secondary types. While the first form has no identifiable trigger, the last one allows for the description of one or more precipitating factors, like drugs, stress, a neurological disease, etc. Arising from N2 sleep or wakeful EEG during SRED episodes and lack of significant polysomnographic variables have been reported (92, 120). Sleepwalking, but also a personal history of eating disorders in patients with SRED support (115), once again, the dual nature of this disorder, and the intricate pathophysiology that could reunite aspects from both sleep disorders and eating disorders. Possible involvement of glucose metabolism dysfunctions was explored in patients with SRED, and a decrease of this metabolism in the cortex during sleep has been suggested (53). As mentioned before, various neurotransmitter dysfunctions have been invoked as potential pathogenetic mechanisms, but they are not supported by good-quality evidence. For example, a decrease in the dopaminergic neurotransmission in SRED has been hypothesized based on the antipsychotic drugs’ mechanism of action, while the enhancement of GABA-ergic activity has been suggested based on the Z-drugs and benzodiazepine effects (45, 73). The presumed arousal of regions within the reward system and the co-occurrence of SRED and RLS have also supported dopamine circuitry dysregulation (122). A role for serotonergic dysregulation exists if the possible impact of Z-drugs and antipsychotics on this system is considered (45, 53, 73). Genetic factors may be involved in the SRED pathogenesis, as suggested by isolated data (14, 125).

Other factors have been involved in the pathogenesis of sleep disorders, and leptin, a protein hormone produced by fat cells, has a circadian rhythm modulated by sleep (145). Although not specifically related to SRED pathogenesis, variations in leptin blood levels are an interesting topic for further exploration, because it is involved in the regulation of appetite. Short duration of sleep, sleep fragmentation, and OSA have been associated with contradictory findings on leptin levels. However, sustained insufficient sleep induced a lower fasting leptin blood level, contributing to increased appetite, obesity and OSA, and CPAP may decrease hyperleptinemia, suggesting leptin could be a biomarker for treatment efficacy in patients with OSA (145). Further exploration of the relationship between leptin blood levels and sleep disturbances is also supported by (a) higher leptin and insulin levels in hospital nurses working the night shift, and eating at night was associated with these biological variations; (b) chronic insufficient sleep in children was associated with lower leptin blood level, especially in 7-year old girls with greater adiposity and male adolescents (each 1-hour decrease of sleep duration was associated with 0.06 decrease of log leptin); (c) increased sleep duration in children of 8–11 years of age (N=37 participants monitored for three weeks) determined lower food intake, lower fasting leptin blood levels, and lower weight (145–148).

Cortical arousals are considered complex phenomena occurring within the central nervous system that involve not only the cortex but also the thalamus, brainstem, and spinal cord (149, 150). OSA, as well as periodic limb movement disorder (PLMD), may lead to an increased frequency of arousals during sleep and a rise in the frequency of parasomnias, SRED included. OSA was associated with both increased sleep fragmentation and increased homeostatic sleep pressure. The fragmentation of NREM sleep is an important pathogenetic pathway for triggering SRED onset and maintaining its occurrence in the case of OSA through chronic intermittent airway collapse (44). PLMD is also associated with frequent arousals, according to polysomnographic studies, being suggested that an underlying arousal disorder may exist in these patients, producing a periodic activation and deactivation of the cerebral cortex (151). Synchronized arousals in PLMD are responsible for sleep fragmentation, which explains insomnia, daytime sleepiness, and fatigue (152). Video-polysomnographic investigations detected in these patients nighttime body movements and sleep talking only during non-REM sleep, and these phenomena were correlated with periodic leg movement-induced arousals (149, 152). The paucity of data regarding the PLMD in relation to SRED is possible due to the fewer reports on SRED in general when compared to other NREM disorders, like sleepwalking, sleep talking, or night terrors, for which multiple studies and reviews exist (153–155). Although systematic studies of cortical arousals as a pathogenetic factor in patients with OSA or PLMD and comorbid SRED have not yet been conducted, it could be an important direction for future research, with potentially important therapeutic implications.

The differential diagnosis includes, first of all, the NES, a disorder with a still uncertain nosological status, but with clinical criteria defined by the DSM-5TR (12). It is important to note that no drug-induced NES exists, unlike SRED (12). Although there is controversy regarding the NES and SRED as distinct disorders (130), both disorders have specified sets of diagnosis criteria. Other clinical entities that should be included in the differential diagnosis of SRED are KLS, BED, bulimia nervosa, and dissociative disorders. Also, the presence of NDNE is important to consider to avoid over-pathologizing nocturnal eating.

Few epidemiological data on SRED have been identified in the literature, but it appears this disorder is more frequently detected in women, individuals undergoing treatment with SSRIs and benzodiazepines, and patients with eating disorders, depression, and obesity (50, 80, 86, 114). There is a long duration of SRED evolution until the diagnosis is made, varying from 12 to 39 years, or even more (114, 131).

Few instruments for screening parasomnias exist, and, while none of these are specific for SRED, they may be useful in differentiating SRED from NES or other related disorders (80, 120, 134, 137).

Treatment of SRED has as its main direction the removal of triggers and is based largely on clinicians’ experience (14). Most data retrieved in the literature are derived from case reports, case series, and small trials of limited quality. SSRIs, topiramate, and clonazepam are the most supported therapeutic recommendations (90). Combining pramipexole and clonazepam, carbidopa/l-dopa and codeine, phentermine and topiramate ER, bupropion + l-dopa + trazodone are also explored drug associations for SRED (63, 64, 95, 104). Agomelatine, pramipexole as monotherapy, suvorexant, and ramelteon benefit from favorable results in case reports or small studies (95, 103, 105, 106). Psychotherapy, hypnotherapy and CBT included, may be useful for patients with SRED, but there is an obvious need for larger trials to support its efficacy (107–109). A limited offering of food before going to bed could be useful, according to two case reports (83, 92).

As a limitation of this systematic review, the quality of sources was assessed by only one author. Due to the same reason, the selection of the papers for inclusion may be subjected to biases. However, adhering to the PRISMA guidelines for systematic reviews was intended to limit the potential impact of such limitations. Another constraint refers to the high percentage of case reports out of the entire body of research explored, which is inherently low quality data. No meta-analysis could be performed due to the extremely low number of controlled trials dedicated to SRED. As a strength of the review, its approach to all the main dimensions used in describing any mental or organic disorder, i.e., the epidemiology, risk factors, differential diagnosis, pathogenesis, methods of evaluation, and treatment, differentiates it from other reviews on this topic.

Future directions in the domain of SRED should target three directions: (1) finding clinical and preclinical models of this pathology, based on neurobiological and neuro-imagistic data, but also able to include evidence from genetic and epidemiological studies; also, investigating multiple dimensions of the pathophysiology involved in the genesis of other eating or sleep disorders (156–161) is expected to offer new insights into the origins of SRED; (2) prospective trials dedicated to the treatment of SRED, using pharmacological agents, psychotherapy, or combined treatment; (3) increasing the awareness of physicians, starting from GPs to the mental health specialists, through educational programs, about the existence of SRED, NES, and related disorders.

## 5 Conclusion

SRED is a complex pathology situated at the crossroads between eating disorders and sleep disorders, integrating elements from both nosological categories but maintaining its individuality. Because the level of awareness during the eating episodes could not always be adequately assessed, the screening for both NES and SRED, when nocturnal eating behaviors appear, is granted. Also, using other instruments for daytime eating disorders and parasomnias, in general, is recommended whenever SRED is suspected. Regarding the use of drugs in patients with sleep disorders, it is important to note that several drugs have the ability to induce SRED, while also being useful in treating this condition. It is expected that further research on the pathophysiology of SRED will help clinicians understand the particularities of drugs and vulnerability factors interaction, thus allowing them to elaborate adequate preventive strategies and therapeutic approaches. Also, since SRED requires an interdisciplinary approach, it is important to mention that this pathology should be the focus of clinicians and researchers working in the fields of sleep medicine, neurology, psychiatry, pulmonology, endocrinology, and related areas of medicine, but also of clinical psychologists and psychotherapists. By creating multidisciplinary teams to investigate SRED, appropriate detection strategies and integrated case management guidelines for a complex pathology like SRED could be constructed.

## Data availability statement

The original contributions presented in the study are included in the article/**Supplementary Material**. Further inquiries can be directed to the corresponding author.

## Author contributions

OV: Writing – review & editing, Writing – original draft, Visualization, Validation, Supervision, Software, Resources, Project administration, Methodology, Investigation, Funding acquisition, Formal analysis, Data curation, Conceptualization.
