# Supplementary material for: Current evidence and future perspectives in the exploration of sleep-related eating disorder–a systematic literature review
Source: Front Psychiatry. 2024 May 30;15:1393337. doi: 10.3389/fpsyt.2024.1393337 (PMC11169790; doi:10.3389/fpsyt.2024.1393337)
Supplement: Supplementary file 2 [file Table_2.docx]

**Table S2. JBI appraisal of the data quality**

| **Reference** | **Type of Research** | **JBI Criteria** | | | | | | | | | | | | | **Score (percentage)** |
| --- | --- | --- | --- | --- | --- | --- | --- | --- | --- | --- | --- | --- | --- | --- | --- |
|  |  | 1 | 2 | 3 | 4 | 5 | 6 | 7 | 8 | 9 | 10 | 11 | 12 | 13 |  |
| [29] | SR | Y | Y | Y | Y | Y | ? | N | N | N | N | N |  |  | 5 (45.4%) |
| [30] | CR | Y | N | Y | N | Y | Y | ? | ? |  |  |  |  |  | 4 (50%) |
| [31] | NR | Y | Y | N | ? | ? | Y |  |  |  |  |  |  |  | 3 (50%) |
| [32] | NR | Y | N | ? | ? | ? | Y |  |  |  |  |  |  |  | 1 (16.6%) |
| [33] | PS | Y | Y | ? | N | ? | Y | Y | Y | N/A |  |  |  |  | 5 (55.5%) |
| [34] | PS | Y | Y | ? | N | Y | Y | Y | Y | N/A |  |  |  |  | 6 (66.6%) |
| [35] | PS | Y | Y | ? | N | Y | N | Y | Y | N/A |  |  |  |  | 5 (55.5%) |
| [36] | PS | Y | ? | Y | N | N | Y | Y | Y | N/A |  |  |  |  | 5 (55.5%) |
| [37] | CS | N | Y | Y | N | N | Y | Y | N | Y | Y |  |  |  | 6 (60%) |
| [38] | CS | N | N | N | ? | N | Y | Y | Y | N | N/A |  |  |  | 4 (40%) |
| [39] | CR | Y | N | ? | Y | Y | Y | Y | Y |  |  |  |  |  | 6 (75%) |
| [40] | CR | Y | N | Y | Y | ? | ? | N | Y |  |  |  |  |  | 4 (50%) |
| [41] | CS | Y | N | N | Y | Y | Y | ? | N | N | N/A |  |  |  | 4 (40%) |
| [42] | CR | Y | ? | Y | Y | N | N | N | Y |  |  |  |  |  | 4 (50%) |
| [43] | CR | Y | ? | Y | Y | N | Y | N | Y |  |  |  |  |  | 5 (62.5%) |
| [44] | CR | Y | N | N | N | Y | N | Y | Y |  |  |  |  |  | 4 (50%) |
| [45] | CR | Y | N | Y | N | N | Y | Y | Y |  |  |  |  |  | 5 (62.5%) |
| [46] | CR | Y | N | Y | N | N | N | N | Y |  |  |  |  |  | 3 (37.5%) |
| [47] | CR | Y | N | N | N | N | N | N | Y |  |  |  |  |  | 2 (25%) |
| [48] | CR | Y | N | Y | N | N | Y | N | Y |  |  |  |  |  | 4 (50%) |
| [49] | CR | Y | Y | N | N | Y | ? | Y | Y |  |  |  |  |  | 5 (62.5%) |
| [50] | CR | Y | Y | Y | Y | ? | ? | Y | Y |  |  |  |  |  | 6 (75%) |
| [24] | CS | Y | Y | Y | N | ? | Y | Y | Y | N | Y |  |  |  | 7 (70%) |
| [51] | CS | Y | Y | Y | N | ? | Y | Y | Y | N | Y |  |  |  | 7 (70%) |
| [52] | CR | N | N | N | N | Y | N | Y | Y |  |  |  |  |  | 3 (37.5%) |
| [53] | CR | N | N | Y | Y | Y | N | N | Y |  |  |  |  |  | 4 (50%) |
| [54] | CR | N | N | Y | Y | Y | N | N | Y |  |  |  |  |  | 4 (50%) |
| [55] | CR | N | N | Y | N | Y | N | N | Y |  |  |  |  |  | 3 (37.5%) |
| [56] | CR | Y | N | N | N | Y | N | Y | Y |  |  |  |  |  | 4 (50%) |
| [57] | CR | N | N | Y | N | N | ? | Y | Y |  |  |  |  |  | 4 (50%) |
| [58] | CS | N | ? | ? | N | N | Y | Y | Y | N | N/A |  |  |  | 3 (30%) |
| [59] | CR | N | N | ? | ? | N | Y | Y | Y |  |  |  |  |  | 3 (37.5%) |
| [60] | CR | N | Y | ? | ? | Y | ? | Y | Y |  |  |  |  |  | 4 (50%) |
| [61] | CR | Y | N | Y | N | Y | N | N | Y |  |  |  |  |  | 4 (50%) |
| [62] | CR | N | N | Y | N | ? | Y | Y | Y |  |  |  |  |  | 4 (50%) |
| [63] | CR | N | N | Y | N | Y | ? | ? | Y |  |  |  |  |  | 3 (37.5%) |
| [64] | CR | Y | N | ? | N | ? | Y | Y | Y |  |  |  |  |  | 4 (50%) |
| [65] | CC | Y | Y | ? | Y | ? | ? | N | Y | Y | Y |  |  |  | 6 (60%) |
| [66] | EO | Y | ? | ? | ? | Y | ? |  |  |  |  |  |  |  | 2 (33.3%) |
| [67] | PS | Y | ? | ? | N | Y | Y | Y | Y | ? |  |  |  |  | 5 (55.5%) |
| [23] | CC | ? | ? | Y | Y | ? | N | N | Y | Y | Y |  |  |  | 5 (50%) |
| [68] | PS | N | ? | ? | N | ? | Y | ? | Y | N/A |  |  |  |  | 2 (22.2%) |
| [69] | PS | Y | Y | ? | Y | Y | Y | Y | Y | ? |  |  |  |  | 7 (77.7%) |
| [70] | CR | ? | N | ? | Y | Y | ? | Y | Y |  |  |  |  |  | 4 (50%) |
| [71] | CS | N | Y | Y | N | N | ? | ? | ? | Y | N/A |  |  |  | 3 (30%) |
| [72] | CC | Y | Y | Y | Y | Y | N | N | Y | Y | Y |  |  |  | 7 (70%) |
| [73] | CC | Y | Y | Y | Y | Y | N | N | Y | ? | Y |  |  |  | 7 (70%) |
| [74] | CS | ? | Y | ? | N | N | ? | Y | Y | N | N/A |  |  |  | 3 (30%) |
| [75] | CR | N | N | Y | N | Y | N | Y | Y |  |  |  |  |  | 4 (50%) |
| [10] | EO | Y | ? | Y | ? | Y | ? |  |  |  |  |  |  |  | 3 (50%) |
| [11] | EO | Y | ? | ? | Y | Y | ? |  |  |  |  |  |  |  | 3 (50%) |
| [88] | NR | Y | ? | ? | Y | Y | ? |  |  |  |  |  |  |  | 3 (50%) |
| [104] | NR | Y | N | ? | N | ? | Y |  |  |  |  |  |  |  | 2 (33.3%) |
| [76] | RCT | N | ? | ? | Y | ? | ? | Y | Y | Y | Y | Y | Y | ? | 7 (53.8%) |
| [86] | CR | N | N | Y | Y | ? | Y | ? | Y |  |  |  |  |  | 4 (50%) |
| [105] | EO | Y | ? | Y | ? | Y | ? |  |  |  |  |  |  |  | 3 (50%) |
| [106] | CS | Y | Y | ? | ? | ? | ? | Y | Y | N | ? |  |  |  | 4 (40%) |
| [107] | CR | Y | ? | Y | N | ? | ? | Y | Y |  |  |  |  |  | 4 (50%) |
| [108] | RCT | Y | Y | Y | Y | Y | ? | ? | Y | Y | Y | Y | Y | Y | 11 (84.6%) |
| [109] | CS | N | N | ? | ? | ? | Y | ? | Y | Y | Y | Y | Y | ? | 6 (461%) |
| [110] | CS | N | Y | Y | N | N | Y | ? | ? | N | N/A |  |  |  | 3 (30%) |
| [111] | CR | ? | N | Y | N | Y | N | Y | Y |  |  |  |  |  | 4 (50%) |
| [112] | CS | Y | ? | Y | Y | ? | Y | ? | Y | N | Y |  |  |  | 6 (60%) |
| [113] | CR | N | ? | Y | N | Y | Y | Y |  |  |  |  |  |  | 4 (50%) |
| [114] | CS | Y | ? | Y | N | N | N | Y | Y | N | N/A |  |  |  | 4 (40%) |
| [115] | CR | Y | Y | Y | N | ? | Y | ? | Y |  |  |  |  |  | 5 (62.5%) |
| [116] | CS | ? | Y | Y | N | N | N | Y | Y | N | N/A |  |  |  | 3 (30%) |
| [117] | CR | ? | Y | Y | Y | Y | ? | ? | Y |  |  |  |  |  | 5 (62.5%) |
| [118] | CS | Y | ? | ? | Y | Y | Y | Y | Y | Y | Y |  |  |  | 8 (80%) |
| [119] | CS | Y | ? | Y | N | ? | N | ? | Y | N | ? |  |  |  | 3 (30%) |
| [120] | CR | Y | N | Y | N | ? | Y | ? | Y |  |  |  |  |  | 4 (50%) |
| [121] | CHS | N/A | N/A | Y | N | N | Y | Y | Y | Y | N | Y |  |  | 6 (54.5%) |
| [122] | NR | Y | ? | Y | Y | ? | Y |  |  |  |  |  |  |  | 4 (66.6%) |

CC= case-control study; CHS= cohort study; CR= case report; CS= case series; EO= expert opinion; NR= narrative review; PS= prevalence study; RCT= randomized controlled trial;

SR= systematic review; Y= yes; N= no; ?= unclear; N/A= not applicable

**JBI criteria used** [36-41]: **CC**: Were the groups comparable other than the presence of disease in cases of the absence of disease in controls? Were cases and controls matched appropriately? Were the same criteria used for identification of cases and controls? Was exposure measured in a standard, valid and reliable way? Was exposure measured in the same way for cases and controls? Were confounding factors identified? Were strategies to deal with confounding factors stated? Were outcomes assessed in a standard, valid and reliable way for cases and controls? Was the exposure period of interest long enough to be meaningful? Was appropriate statistical analysis used?; **CHS**: Were the two groups similar and recruited from the same population? Were the exposure measured similarly to assign people to both exposed and unexposed groups? Was the exposure measured in a valid and reliable way? Were confounding factors identified? Were strategies to deal with confounding factors stated? Were the groups/participants free of the outcome at the start of the study (or at the moment of exposure)? Were the outcomes measured in a valid and reliable way? Was the follow-up time reported and sufficient to be long enough for outcomes to occur? Was the follow up complete, and if not, were the reasons to loss to follow-up described and explored? Were strategies to address incomplete follow up utilized? Was appropriate statistical analysis used? **CR**: Were patients’s demographic characteristics clearly described? Was the patient’s history clearly described and presented as a timeline? Was the current clinical condition of the patient on presentation clearly described? Were diagnostic tests or assessment methods and the results clearly described? Was the intervention(s) or treatment procedure(s) clearly described? Was the post-intervention clinical condition clearly described? Were adverse events (harms) or unanticipated events identified and described? Does the case report provides takeaway lessons? **CS**: Were there clear criteria for inclusion in the case series? Was the condition measured in a standard, reliable way for all participants included in the case series? Were valid methods used for identification of the condition for all participants included in the case series? Did the case series have consecutive inclusion of participants? Did the case series have complete inclusion of participants? Was there clear reporting of clinical information of the participants? Were the outcomes or follow up results of cases clearly reported? Was there clear reporting of the presenting site(s)/clinic(s) demographic information? Was statistical analysis appropriate? **EO**: Is the source of the opinion clearly identified? Does the source of opinion have standing in the field of expertise? Are the interests of the relevant population the central focus of the opinion? Does the opinion demonstrate a logically defended argument to support the conclusions drawn? Is there a reference to the extant literature? Is any incongruence with the literature/sources logically defended?; **NR**: Is the generator of the narrative review a credible or appropriate source? Is the relationship between the text and its context explained? Does the narrative present the events using a logical sequence so the reader or listener can understand how it unfolds? Do you, as a reader or listener of the narrative, arrive at similar conclusions to those drawn by the narrator/ Do the conclusions flow from the narrative account? Do you consider this account to be a narrative?; **PS**: Was the sample frame appropriate to address the target population? Were study participants sampled in an appropriate way? Was the study sample adequate? Were the study subjects and setting described in detail? Was the data analysis conducted with sufficient coverage of the identified sample? Were valid methods used for the identification of the condition? Was the condition measured in a standard, reliable way for all participants? Was there appropriate statistical analysis? Was the response rate adequate, and if not, was the low response rate managed appropriately? **RCT**:Was true randomization used for assignment of participants to study groups? Was allocation to treatment groups concealed? Were treatment groups similar at the baseline/ Were participants blind to treatment assignment? Were outcomes assessors blind to treatment assignment? Were treatment groups treated identically other than the intervention of interest? Was follow-up complete, and if not, were differences between groups in terms of their follow-up adequately described and analyzed? Were participants analyzed in the groups to which they were randomized? Were outcomes measured in the same way for treatment groups? Were outcomes measured in a reliable way? Was appropriate statistical analysis used? Was the trial design appropriate, and any deviations from the standard RCT design accounted for in the conduct and analysis of the trial? **SR**: Is the review question clearly and explicitly stated? Were the inclusion criteria appropriate for the review question? Was the search strategy appropriate? Were the sources and resources used to search for studies adequate? Were the criteria for appraising studies appropriate? Was critical appraisal conducted by two or more reviewers independently? Were there methods to minimize errors in data extraction? Were the methods used to combine studies appropriate? Was the likelihood of publication bias assessed? Were recommendations for policy and/or practice supported by the reported data? Were the specific directives for new research appropriate?
